# Saponins: Research Progress and Their Potential Role in the Post-COVID-19 Pandemic Era

**DOI:** 10.3390/pharmaceutics15020348

**Published:** 2023-01-20

**Authors:** Daniel Mieres-Castro, Freddy Mora-Poblete

**Affiliations:** Institute of Biological Sciences, University of Talca, 1 Poniente 1141, Talca 3465548, Chile

**Keywords:** saponin, COVID-19, antiviral, anti-inflammatory, antithrombotic, immunostimulatory, adjuvant

## Abstract

In the post-COVID-19 pandemic era, the new global situation and the limited therapeutic management of the disease make it necessary to take urgent measures in more effective therapies and drug development in order to counteract the negative global impacts caused by severe acute respiratory syndrome coronavirus 2 (SARS-CoV-2) and its new infectious variants. In this context, plant-derived saponins—glycoside-type compounds constituted from a triterpene or steroidal aglycone and one or more sugar residues—may offer fewer side effects and promising beneficial pharmacological activities. This can then be used for the development of potential therapeutic agents against COVID-19, either as a therapy or as a complement to conventional pharmacological strategies for the treatment of the disease and its prevention. The main objective of this review was to examine the primary and current evidence in regard to the therapeutic potential of plant-derived saponins against the COVID-19 disease. Further, the aim was to also focus on those studies that highlight the potential use of saponins as a treatment against SARS-CoV-2. Saponins are antiviral agents that inhibit different pharmacological targets of the virus, as well as exhibit anti-inflammatory and antithrombotic activity in relieving symptoms and clinical complications related to the disease. In addition, saponins also possess immunostimulatory effects, which improve the efficacy and safety of vaccines for prolonging immunogenicity against SARS-CoV-2 and its infectious variants.

## 1. Introduction

Saponins are compounds widely distributed in nature. They are mainly found in higher plants as secondary metabolites and are reported in almost 100 families [1,2]. These glycoside-type compounds are constituted from a triterpene or steroidal aglycone known as sapogenin (the hydrophobic part) and one or more sugar residues (the hydrophilic part), which are linked through ester or ether linkages [2,3,4]. Most triterpenoid saponin aglycones have a pentacyclic oleanane, ursane, hopane, or dammarane-type structure. While most aglycones of steroidal saponins have a spirostane, glycoalkaloid, and furostane-type structure [2,3,4]. The amphiphilic nature of these compounds allows them to act as surfactants and produce soapy foams when shaken with water. This characteristic has led to different saponin-rich plants being used, traditionally, as natural soaps and detergents [1,2,3,4]. In plants, saponins are present in various organs (roots, rhizomes, stems, bark, leaves, seeds, and fruits), which play a defensive role against bacteria, fungi, viruses, parasites, and insects [5,6,7].

Plants and plant-derived products that contain saponins are important in human and animal nutrition [5,6,7,8]. Saponins consumed through the diet (vegetables, supplements, or extracts) undergo extensive gastrointestinal metabolism, presenting low absorption at the gastric and small intestine levels, thereby allowing them to reach the large intestine where they are hydrolyzed via the colonic microbiota and, thus, are subsequently absorbed [9,10,11]. The colonic transformation of saponins allows the release of sapogenins (aglycones), thereby influencing their bioavailability to be lower or higher when compared to the precursor saponin. This is because changes are produced in the chemical properties of solubility after being released from the sugar residues [9,10,11]. In general, the bioavailability of saponins is low and is explained by different factors that modulate absorption, either before (solubility, automycellation properties, chemical modifications, gastric transformation, and colonic fermentation) or during (membrane permeability, efflux transporters, and specific chemical form) cellular uptake [11]. Therefore, the beneficial biological effects of saponins in the body depend not only in their chemical structure, but also in their bioavailability [5,6,7,8,9,10,11]. In this context, multiple studies have reported that a wide variety of saponins, which were obtained from medicinal plants or species of agricultural importance, exhibit different biological and pharmacological properties [1,3,8,9,10,11,12,13,14,15,16]. These properties include membrane permeabilization [17,18], anticancer [19,20] antithrombotic [21], immunostimulant [22], hypoglycemic [23,24], hypocholesterolemic [25], anti-inflammatory [26], antibacterial [27], antifungal [28], antiparasitic [29], and antiviral [30] effects. Various plant species are considered of great agronomic importance due to their high content of saponins, which have been found to have multiple beneficial applications for human health. As such, they are being used in the pharmaceutical industry for the production of drugs, adjuvants for vaccines, food supplements, flavor modifiers, or sweeteners; as well as in the cosmetic industry, as emulsifiers, precursors for hormone synthesis, among others [1,3,8,9,10,11,12,13,14,15]. Examples of natural sources rich in saponins include plants used as drug sources (such as ginseng, horse chestnut, cassava, quinoa, licorice, caltrop, chaihu, or quillay) and also crop plants (such as legumes and oats) [1,2].

In the post-COVID-19 pandemic era, the new global scenario makes it necessary to take urgent measures in order to counteract the negative impacts caused by SARS-CoV-2 and its novel infectious variants [31]. In fact, the pandemic waves, which were caused by the different variants of the virus, have had a catastrophic effect worldwide with more than 6.6 million deaths, becoming the most important global health crisis since the era of the 1918 influenza pandemic [32,33]. Current standard operating procedures that are in place to control COVID-19 infection include early diagnosis (molecular diagnosis by RT-qPCR test or nasopharyngeal or salivary antigen test) in order to reduce the chances of secondary infection, followed by isolation of cases (quarantine), as well as supportive care through preventive (vaccines) and therapeutic (retroviral and anti-inflammatory) strategies [33,34,35]. Although, currently, the anti-SARS-CoV-2 pharmacological strategy is undertaken with antivirals, antiretrovirals, or antimalarials (e.g., nirmatrelvir, ritonavir, molnupiravir, chloroquine, hydroxychloroquine, etc.), as well as different authorized vaccines that are available that allow the generation of immunity against the virus, there is, to date, still limited therapeutic management of this viral infection due to the appearance of different viral variants [34,35,36]. Therefore, current procedures have focused on developing and testing new and more effective drugs, or in the reuse of existing drugs against possible therapeutic targets of the virus [32,34]. In this context, and when compared to synthetic drugs, active compounds from natural sources—including saponins and plant sources rich in them—offer fewer side effects and may have promising effects [34,37]. This can be in the form either as agents with anti-SARS-CoV-2 exerting effects on pharmacological targets of the virus [38,39,40,41] as therapy, as a complement to the conventional treatment of the symptoms of the COVID-19 disease and its different clinical complications [42,43,44,45,46,47,48], or as an adjuvant for more efficient vaccine development [36,49].

The main objective of this review was to examine the primary and current evidence on the therapeutic potential of plant-derived saponins against the COVID-19 disease. This was achieved by focusing on those studies that highlight the potential use of saponins as a treatment against SARS-CoV-2. Based on current scientific evidence, this review addresses the potential of saponins and their natural sources in the treatment and prevention of COVID-19.

## 2. Materials and Methods

The databases used in this critical review included Google Scholar, PubMed, ScienceDirect, SciFinder, Scopus, and SciELO. In addition, the literature review was carried out according to Liberati et al. [50]. The keywords used for the search were: “saponins + COVID-19”, “saponins + clinical trial + COVID-19”, “saponins + pharmacological effects + COVID-19”, “saponins + anti-SARS-CoV-2 activity”, “saponins + cytokine storm + COVID-19”, “saponins + platelet aggregation + thrombosis + COVID-19”, and “saponins + adjuvant + vaccine + COVID-19”. For this review, current experimental and review articles related to plant-derived saponins, published in peer-reviewed journals until August 2022, were selected. In the first instance, a superficial analysis was carried out (i.e., reading abstracts). Further, articles that were: in languages other than English, theses, and possessed unpublished data, were all excluded. The information collected from the articles was interpreted qualitatively, including an in-depth content analysis. After reviewing the full text, articles that were not related to plant-derived saponins, as well as articles with a limited presentation of findings, were excluded. As an eligibility criterion—in the section that reviews the pharmacological effects of saponins against symptoms and clinical complications of the COVID-19 disease—only those studies that evaluated the effects of saponins within in vivo models or in clinical trials that were associated with inflammation and/or pulmonary coagulopathy were included in the discussion. Selected primary articles were used in order to identify additional related publications. Using this strategy, a total of 150 references were selected for the purposes of analysis and discussion. The articles that have were discussed summarized the potential of plant-derived saponins for the treatment of COVID-19. These articles possessed special emphases on the use of saponin as anti-SARS-CoV-2 agents; as therapy for symptomatology and clinical complications; and as adjuvants in the production of vaccines.

## 3. Results and Discussion

A total of 206 references published until August 2022 were included in the first screening. After examining these, 150 references were incorporated in the discussion of this review, of which 147 were research articles and 3 were websites. Among the selected references, 33 articles were related to the characteristics and generalities of plant-derived saponins (i.e., chemical structure, plant sources, uses, digestion, bioavailability, and general pharmacological effects); 10 articles and 3 websites were related to COVID-19 data (i.e., viral replication cycle of SARS-CoV-2, symptoms, hyperinflammatory and thromboembolic complications, worldwide incidence, and pharmacological strategies for treatment and prevention); 20 articles were related to the anti-SARS-CoV-2 activity of saponins; 43 articles were related to the anti-inflammatory activity of saponins; 8 articles were related to the antiplatelet–antithrombotic activity of saponins; and 32 articles were related to the immunostimulatory activity of saponins.

### 3.1. Antiviral Activity of Saponins

The antiviral capacity of saponins has been proven within multiple viruses [30], including viruses that cause respiratory pathologies, such as the influenza virus [51,52,53,54], human respiratory syncytial virus (RSV) [55], and SARS-CoV-2 [38,39,40,41,56,57,58,59,60,61,62,63]. Different mechanisms of antiviral action have been described, including the interaction with viral envelope proteins and their destruction, the prevention of the virus binding to host cells by damaging virus binding sites, and the coating of viral receptors on host cells [30].

#### 3.1.1. Anti-SARS-CoV-2 Saponins: Structure-Activity Relationship

**Figure 1:**
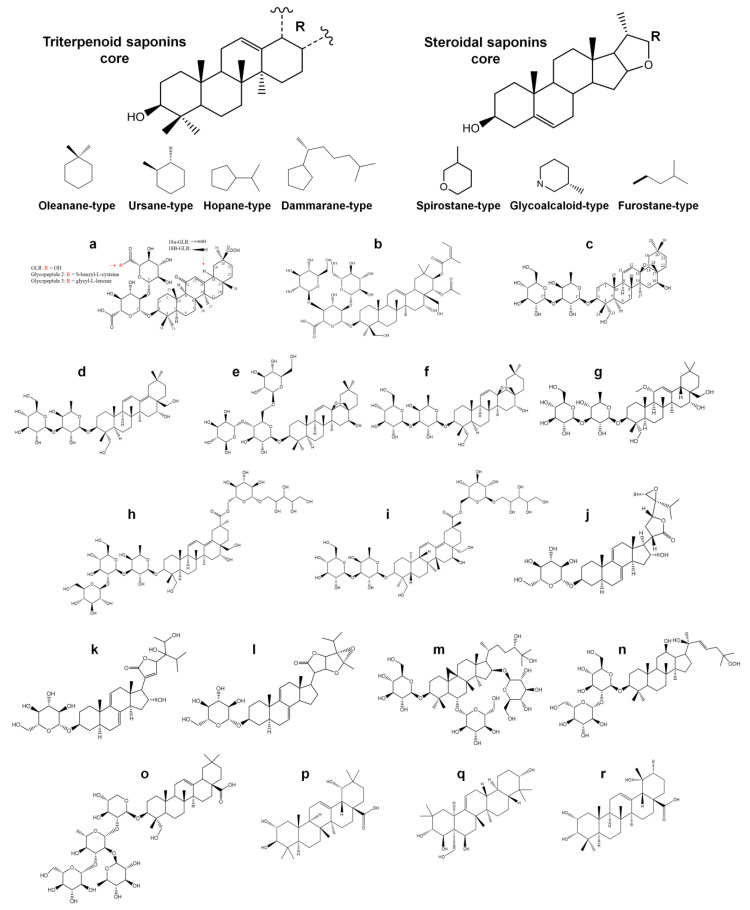
Chemical structure diversity in saponin aglycones and main saponins with anti-SARS-CoV-2 activity. (**a**) Glycyrrhizin; (**b**) β-Escin; (**c**) Saikosaponin A; (**d**) Saikosaponin B2; (**e**) Saikosaponin C; (**f**) Saikosaponin D; (**g**) Saikosaponin B4; (**h**) Saikosaponin U; (**i**) Saikosaponin V; (**j**) Vernonioside A2; (**k**) Vernonioside A4; (**l**) Vernonioside D2; (**m**) XGG-pentahydroxycycloartane; (**n**) Ginsenoside Rg12; (**o**) TPG1; (**p**) arjunic acid; (**q**) Theasapogenil B; and (**r**) euscaphic acid.

There have been only a few studies that have described the antiviral ability of saponins to inhibit the cytopathic effects of SARS-CoV-2 within in vitro cell assay models. Table 1 summarizes the main studies related to the anti-SARS-CoV-2 activity of saponins and their potential effects on different pharmacological targets. Further, Figure 1 shows the chemical structure diversity in saponin aglycones and main saponins with anti-SARS-CoV-2 activity.

##### Glycyrrhizin

Glycyrrhizin (GLR), also called glycyrrhizic acid (GA), is a triterpene saponin isolated mainly from the roots of plants that are of the genus *Glycyrrhiza* [30,42,45]. GLR is a glycoside of glycyrrhetinic acid (GA, sapogenin part) with two glucuronic acid residues (Figure 1a). In its natural form, GLR exists as two epimers, 18α-glycyrrhizin (18α-GLR) and 18β-glycyrrhizin (18β-GLR), both of which are active, although the 18β isomer appears to be more potent than the 18α isomer [42]. This compound is considered a safe natural product, with a long-established use in the food, cosmetic, and pharmaceutical industries [42]. In humans, this drug is administered in oral and intravenous formulations for the treatment of liver diseases, particularly chronic viral hepatitis [42]. This use of GLR is based on multiple studies that have shown its marked antiviral activity against different viruses (i.e., Hepatitis A/B/C viruses, HIV-1 virus, Varicella-zoster virus, Epstein–Barr virus, Chikungunya and Semliki Forest viruses, and dengue virus), including some that are associated with respiratory pathologies (i.e., RSV, Influenza A virus, and Parainfluenza virus type 2), which has led to the proposal of GLR as an alternative (or complementary) drug for the treatment of the COVID-19 infection and its clinical complications [30,42,45]. Cinatl et al. [56] evaluated the antiviral potential of GLR against two clinical isolates (FFM-1 and FFM-2) of coronavirus that were obtained from SARS patients admitted to the clinical center in the University of Frankfurt, Germany. The MTT assay showed that GLR inhibited viral cytopathic effects in Vero cells, reporting a half-maximal effective concentration (EC_50_) of 300 µg/mL for the inhibition of virus replication, but also inhibition of virus adsorption and penetration into cells. The antiviral action was more effective when GLR was administered during and after the adsorption period. The authors, therefore, suggest that GLR stimulated nitric oxide synthase (iNOS) activity in Vero cells. Therefore, this increased the nitric oxide, which, in turn, exerted a toxic effect on the virus. Chen et al. [57] also evaluated the antiviral activity of GLR against 10 clinical isolates from SARS patients from different hospitals in the Hong Kong Special Administrative Region. Using a plaque reduction assay, it was reported that incubation of Vero-E6 cells with GLR during viral infection was able to inhibit viral replication with an EC_50_ of 100 µg/mL. However, the authors emphasized that the rapid metabolism of the drug limits its exposure, thereby not allowing the effective concentration to be reached. This is due to the fact that even when an intravenous dose of 200 mg was administered to patients, the maximum serum level was only 80 µg/mL, which is still below the EC_50_ reported in their in vitro assays. Hoover et al. [58] evaluated the anti-SARS-CoV activity of GLR, and its derivatives, by measuring the cytopathic effect (MTT assay) and viral antigen expression (immunocytochemical staining) on Vero cells. The addition of 2-acetamido-β-D-glucopyranosylamine to the glycoside chain of GLR (Figure 1a) was shown to result in up to a 10-fold increase in anti-SARS-CoV activity (EC_50_ 35 µM for the glycopeptide 2 containing S-benzyl-L-cysteine, and EC50 139 µM for glycopeptide 3 containing glycyl-L-leucine) when compared to GLR (EC_50_ 365 µM). The authors speculated that the insertion of these N-acetylglucosamine residues in the carbohydrate portion of the GLR molecule increases its hydrophilic properties, thereby allowing a better interaction with the carbohydrates that presents the viral spike glycoprotein. This, thus, inhibits the interaction of the virus with the angiotensin-converting enzyme 2 (ACE2); moreover, this, consequently, prevents the entry of the virus into the host cell. On the other hand, those GLR amides and GLR conjugates with two amino acid residues and a free 30-COOH function considerably increased the activity against SARS-CoV (up to 70 times more); however, it also increased the cytotoxicity of compounds, thereby decreasing their selectivity index.

##### Escin

Escin is a natural mixture of triterpene saponins that are extracted from horse chestnut seeds (i.e., *Aesculus hippocastanum*) and whose main active component is β-Escin (Figure 1b). It has been proposed as a possible traditional medicine for the treatment of COVID-19 due to the fact that it has shown a powerful antiviral effect against SARS-CoV, RSV, and herpes simplex virus type 1 (HSV-1), among others [43]. Wu et al. [59] evaluated the anti-SARS-CoV activity of more than 10,000 small molecules, including Escin, showing it to be one of the most active compounds by significantly inhibiting viral replication, with an EC_50_ of 6 μM in Vero E6 cell cultures, pretreated with Escin for 2 h before infection with SARS-CoV, demonstrating its ability to prevent the entry of the virus in the host cell, and thus avoid cytopathic effects.

##### Saikosaponins

Saikosaponins are triterpene saponin glycosides derived from oleanan that may possess different types of aglycones, including: 28-epoxy ether, heterocyclic diene, 12-ene, homocyclic diene, 12-ene-28-carboxylic acid, heterocyclic diene-30-carboxylic acid, and 18-ene. They are usually bound to glucose, furanose, rhamnose, xylose, or pentitol [38,64,65]. Saikosaponins are extracted from different plants that are used in traditional oriental medicine, including *Bupleurum* spp., *Heteromorpha* spp., and *Scrophularia scorodonia* [64,65]. Multiple studies have demonstrated the variety of pharmacological effects that Saikosaponins possess [65], including their potent antiviral effects [38,53,62,63,64,65], which has led to their being proposed as candidates for the treatment of COVID-19 [44]. Chen et al. [38] confirmed the antiviral effect of Saikosaponins A, B2, C, and D (Figure 1c–f, respectively) on human coronavirus (HCoV-229E), evaluating the ability to inhibit the cytopathic effects of binding and viral penetration into MRC-5 cells via a XTT cell-proliferative assay. It was found that all Saikosaponins (A, B_2_, C, and D) had anti-HCoV activity at the concentrations tested, reporting EC_50_ values of 8.6, 1.7, 19.9, and 13.2 μM, respectively. Saikosaponins A and B_2_ did not exhibit cytotoxic effects on MRC-5 cells at concentrations that achieved antiviral activity (50% cytotoxicity concentration (CC_50_) = 228.1 and 383.3 μM, respectively). Saikosaponin B_2_ exhibited the highest antiviral activity (EC50: 1.7 μM) and significantly inhibited viral binding to cells in a dose-dependent manner (89.3% inhibition at 25 μM). Additionally, it blocked viral penetration into cells in a time-dependent manner (81.3% inhibition at 90 min using a concentration of 6 μM). The authors suggested that Saikosaponin B_2_ possibly disrupted viral glycoproteins; thus, it interfered with the early stage of viral replication, such as virus attachment and entry.

#### 3.1.2. Effects of Saponins on SARS-CoV-2 Pharmacological Targets

**Figure 2:**
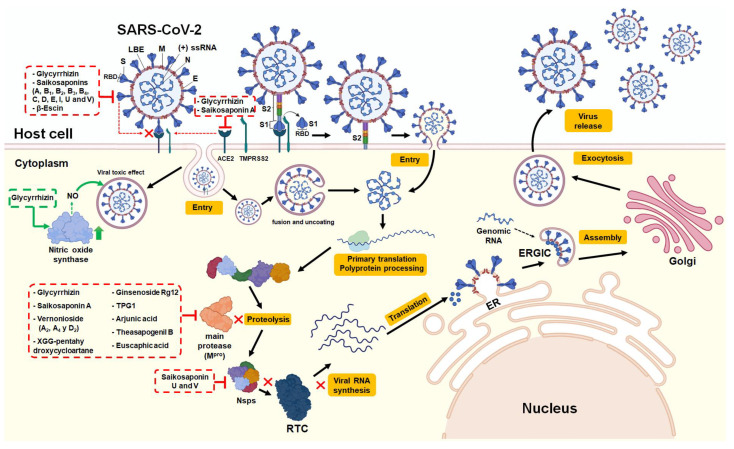
Viral replication cycle of SARS-CoV-2 and the effects of saponins on the viral pharmacological targets. According to Mieres-Castro et al. [66], the structure of SARS-CoV-2 consists of a positive-sense single-stranded RNA genome (+ssRNA), a lipid bilayer envelope (LBE), and different structural proteins including spike or S protein, nucleoprotein (N), membrane protein (M), envelope protein (E), and hemagglutinin esterase (not shown in Figure). The +ssRNA is encapsulated by N, while M and E are incorporated during the viral assembly process. The replication cycle begins with the arrival of the SARS-CoV-2 to the target cell. The S protein binds to ACE2 and its receptor on the host cell; it is, then, cleaved by the cell surface serine protease TMPRSS2, forming two subunits, the S1 subunit containing the receptor-binding domain (RBD), and the S2 subunit containing the peptide for binding to the membrane-bound fusion protein of the host cell. This allows entry of the virus into the host cell, either by the formation of an endosome or by fusion of the viral envelope. After the fusion of the membranes of the virus and the host cell, viral RNA is uncoated and released into the cytoplasm in order to initiate the primary translation of co-terminal polyproteins (pp1a/ab) that perform the viral genome replication. After translation, the homodimeric cysteine protease M^pro^ self-cleaves in order to cleave the polyproteins into Nsps. Different Nsps proteins interact with Nsp12 (also called RNA-dependent RNA polymerase (RdRp)) in order to form the replicase-transcriptase complex (RTC), which synthesizes the full-length viral genome (replication) and subgenomic RNA (transcription). The mRNAs of the viral structural proteins are translated and moved to the endoplasmic reticulum (ER). Genomic RNA is encapsulated with protein N and translocates with structural proteins in the ER-Golgi intermediate compartment (ERGIC) in order to form new virions. Finally, the new viruses are exocytosed from the infected cell and released into the extracellular space in order to infect other cells. The boxes indicate the effects of saponins on the different viral pharmacological targets (red boxes and arrows: inhibition; green boxes and arrows: stimulation). The antiviral mechanisms of action include: inhibiting viral spike glycoprotein and ACE2 in the host cell, thus preventing the binding and entry of the virus; stimulating nitric oxide synthase (iNOS) activity and increasing nitric oxide (NO) in order to cause toxic effects on the virus; and inhibiting M^pro^ (binding to the active site), thus inhibiting the proteolysis of viral polyproteins that are necessary for virus replication, as well as binding to Nsp15 and the inhibition of its activity in the RTC complex for viral RNA synthesis and replication.

One of the most promising anti-SARS-CoV-2 strategies is the development or reuse of drugs that can inhibit proteins that play a central role in the viral replication cycle. This is in spite of whether they are virus proteins—such as the spike glycoprotein (S), type 3C coronavirus protease (also called main protease (M^pro^ or 3CL^pro^)), and non-structural proteins (Nsps)—that make up the replicase-transcriptase complex (RTC). This is in addition to the proteins present in the host cell, such as ACE2 [31]. In this context, in silico molecular docking assays have evaluated the potential use of saponins as inhibitors of these pharmacological targets [39,40,41,60,61,62,63]. The replication cycle of the SARS-CoV-2 and effects of saponins on the viral pharmacological targets are provided in Figure 2.

Chen and Du [60] evaluated the potential of GLR to inhibit human ACE2 and prevent virus–host cell infection. Molecular docking results demonstrated that GLR has the potential to bind ACE2 with a binding free energy of −9.0 kcal/mol, thereby binding to amino acid residues that are close to zinc metallopeptidase (Arg559, Gln388, Arg393, and Asp30) that could regulate the activity of ACE2.

Yan et al. [61] evaluated the inhibitory capacity of the main compounds present in natural medicines (with Chinese patents) on human ACE2 and the SARS-CoV-2 protein M^pro^, which is the key homodimeric cysteine protease that participates in the cleavage of the pp1a/ab polyprotein in order to release the Nsps necessary for virus replication and transcription. Among the compounds evaluated, it was reported that the saponins GLR and Saikosaponin A were molecules with great inhibitory capacity of both pharmacological targets. Both saponins showed higher binding affinity with the active sites of human ACE2 with a binding free energy of −9.9 and −11.0 kcal/mol, respectively, when compared to M^pro^ from SARS-CoV-2, where a binding free energy of −8.9 and −8.8 kcal/mol, respectively, was found. These results suggest that drugs rich in both saponins have great potential for the treatment of COVID-19.

Goswami and Bagehi [62] reported the efficacy of nine Saikosaponins (A, B_1_, B_2_, B_3_, B_4_, C, D, E, and I) in inhibiting the RBD part of the S1 subunit of the S protein of SARS-CoV-2. Most Saikosaponins (except B_2_, C, and I) bind favorably to the RDB region of the S protein. In effect, Saikosaponin B_4_ (Figure 1g) was the best inhibitor among the molecules under study. Further, it presented the greatest interaction with residues Asp428, Arg466, Glu465, and Phe464 of the RBD, with a binding free energy of −13.2 kcal/mol. The authors concluded that Saikosaponin B_4_ can bind to the RBD of the viral spike glycoprotein and thus inhibit the binding of SARS-CoV-2 with the ACE2 receptor of host cells, thereby disabling the first stage of viral infection. Sinha et al. [63] also evaluated the inhibitory potential of 23 different Saikosaponins against the S1 subunit (that contains the RBD) of the S protein and the nonstructural protein 15 (Nsp15) of SARS-CoV-2, an endoribonuclease that is part of the RTC. Further, these are responsible for the synthesis of the complete viral genome (replication) and subgenomic RNA (transcription). Molecular docking studies on both viral proteins revealed that Saikosaponins U and V (Figure 1h,i, respectively) showed better binding with the pharmacological targets under study. This could be attributed to a large number of hydroxyl groups in their structures, either in the sapogenin core or in the side chains. In addition, although both Saikosaponins have an octadecahydropicene ring with a substituted oxane ring, the additional oxane ring present in Saikosaponin U provided a better interaction (H-bond) with the RBD of the S1 subunit of the S protein, positioning itself in the N-terminal domain (residues 319–519) with a binding free energy of −8.4 kcal/mol. The smaller structure of Saikosaponin V allowed it to bind (H-bond) better with amino acids Lys290, Thr341, Tyr343, and Ser294 of the active site of Nsp15, with a binding free energy of −8.4 kcal/mol. These results suggest that Saikosaponins U and V could act by preventing early stages of SARS-CoV-2 infection, such as the union of the virus with the host cell, as well as more advanced stages, such as viral RNA synthesis and replication. 

Ogunyemi et al. [39] evaluated the efficacy of stigmastan-type steroidal saponins from *Vernonia amigdalina*, including Vernonioside A_2_, Vernonioside A_4_, and Vernonioside D_2_ (Figure 1j–l, respectively). Docking analysis revealed that these compounds bound to amino acids located within the active sites, interacting with catalytic residues (catalytic dyad His41 and Cys145) being these potential inhibitors of M^pro^ with a double objective. The calculated binding free energies (−8.6, −8.3, and −8.4 kcal/mol, respectively) corroborated the optimized and static docking analysis. On the other hand, Rehan and Shafiullah [40] evaluated the M^pro^ inhibitory capacity of 60 different saponins from plants used worldwide as traditional medicine. Their findings showed that among the most promising saponins was 3-O-β-D-Xylopyranosyl-6-O-β-D-glucopyranosyl-16-O-β-D-glucopyranosyl-3β,6α,16β,24(S)-25-pentahydroxycycloartane (XGG-pentahydroxycycloartane; Figure 1m) isolated from the roots of *Astragalus brachycalyx* (a legume species from West Asia), which presented the best binding free energy of −11.9 kcal/mol and interacted with residues Arg4, Gln127, Ser139, Glu290, Lys5, and Tyr126 of the M^pro^. Other promising saponins from Asian plants were Ginsenoside Rg12 (Figure 1n) isolated from the roots of *Panax ginseng*, and hederagenin-3-*O*-*β*-D-glucopyranosyl-(1→3)-*α*-L-rhamnopyranosyl-(1→2)-*α*-L-rhamnopyranosyl-(1→2)-*α*-L-arabinopyranoside (TPG1, Figure 1o) isolated from the leaves of *Trevesia palmata*, both with a binding free energy of −10.9 kcal/mol, which bound to residues Thr199 and Leu286 of M^pro^. The binding affinity of these saponins was better than those obtained for drugs used for the treatment of COVID-19, including hydroxychloroquine (−4.6 kcal/mol), chloroquine (−5.6 kcal/mol), and nelfinavir (−7.6 kcal/mol). Falade et al. [41] evaluated the inhibition capacity of different triterpene saponins. Among them, arjunic acid, teasapogenol B, and euscaphic acid (Figure 1p–r, respectively), were found to have the ability to form hydrogen bonds, alkyl, and pi-alkyl interactions with different amino acids of the allosteric pocket of SARS-CoV-2 M^pro^, including Leu286 and Leu287, with binding free energies of −8.1, −8.1 and −8.0 kcal/mol, respectively. Based on their results, the authors suggest the use of these saponins for future research against SARS-CoV-2. This is suggested as they have even better pharmacokinetic and bioavailability properties (i.e., binding energies, ADMET profile, drug similarity, oral bioavailability properties, PASS properties, bioactivity, mode of binding, and molecular interactions with the target receptor), when compared with the native M^pro^ ligand inhibitor (N3 inhibitor; −5.6 kcal/mol), as well as with antiviral drugs used in the management of this disease, such as Remdesivir (−7.6 kcal/mol) and Dexamethasone (−7.7 kcal/mol).

### 3.2. Pharmacological Effects of Saponins against Symptoms and Clinical Complications of the COVID-19 Disease

**Figure 3:**
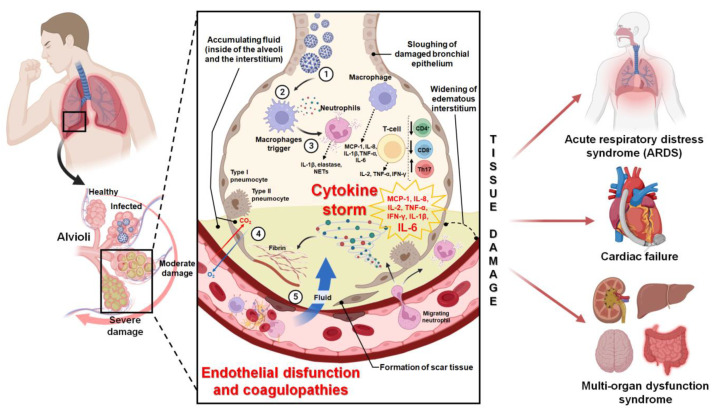
The pathophysiological mechanism of COVID-19 related to clinical complications, such as cytokine storm and thromboembolic coagulopathies. According to Ortega-Paz et al. [68] and Bhaskar et al. [69] the pathophysiological mechanism is characterized by: (**1**) The viral binding of SARS-CoV-2 to the ACE2 receptor present in type II pneumocytes and vascular endothelial cells of the pulmonary alveoli; (**2**) immune cells, including macrophages, that identify the virus, become activated and produce acute response chemokines and cytokines; (**3**) chemokines and cytokines that attract and activate more immune cells, in particular the activation of neutrophils, macrophages, and Th17 cells (and cause the downregulation of CD4^+^ and CD8^+^ T cells) and that causes an exacerbated increase in the production of proinflammatory cytokines, thereby creating a cycle of inflammation or a hyperinflammatory response, such as the cytokine storm. This is then characterized by a marked increase in the monocyte chemoattractant protein-1 (MCP-1), interleukin (IL)-8, IL-2, tumor necrosis factor alpha (TNF-α), Interferon gamma (IFN-γ), IL-1β and in particular IL-6; (**4**) hyper-elevated proinflammatory cytokines could promote and cause cell death, not only of pneumocytes, but also of the endothelial cells of adjacent blood vessels, thereby causing the release of tissue factors (TF), the exposure of subendothelial components (Von Willebrand factor (VWF) and collagen), and fibrin formation. These events stimulate endothelial dysfunction and coagulation (extrinsic and intrinsic pathways), which leads to intravascular thrombosis and, finally, to the development of thromboembolic coagulopathies; (**5**) weakened and damaged vascular endothelium allows protein-rich fluid to leak and fill the lung cavities (into the alveoli and alveolar interstitium), leading to the damage and death of the bronchial epithelium, formation of scar tissue, and failure of respiratory system (decreased gas exchange), which leads to different pathologies and clinical complications. These are also initially generated at the pulmonary level, but which later led to the heart and other tissues of the body causing multi-organ dysfunction syndrome and death.

Most patients with COVID-19 will experience mild to moderate respiratory illness and recover without the need for special treatment since the usual symptoms are fever, chills, dry cough, sputum production, fatigue, lethargy, arthralgia, myalgia, headache, dyspnea, nausea, vomiting, anorexia, and diarrhea [31,33]. However, older adults or people with associated chronic diseases (such as obesity, hypertension, diabetes, thrombotic diseases, chronic respiratory diseases, and cancer) are more likely to develop different serious clinical complications, such as acute hyperinflammatory conditions or thrombotic coagulopathies, which begin in the lung tissue, but spread to different organs of the body, thereby causing multi-organ dysfunction syndrome and death [33,67,68,69] In this context, multiple studies have evaluated the effect of saponins within in vivo models or clinical trials that are associated with acute inflammation and thromboembolic coagulopathies in lung tissue. This has led to the proposing of the potential use of different saponins (triterpenoid and steroidal), as well as extracts that are rich in these, either in therapy or as a complement to the conventional treatment of the COVID-19 disease and its different clinical complications [42,43,44,45,46,47,48]. The pathophysiological mechanism of COVID-19 related to clinical complications, such as cytokine storm and thromboembolic coagulopathies are provided in Figure 3.

#### 3.2.1. Anti-Inflammatory Activity of Saponins and Their Potential Effects against the Cytokine Storm

In critical patients infected with SARS-CoV-2, a condition known as “cytokine storm or hypercytokinemia” has been associated with the disease. This is an acute hyperinflammatory response that results from a systemic spread of the initial inflammatory response, which is generated in the lungs as a result of damage and cell death caused by the virus in pneumocytes and endothelial cells. This process generates the activation of macrophages, neutrophils, and Th17 lymphocytes, as well as results in a downregulation of CD4^+^ and CD8^+^ T cells, thereby promoting a dramatic increase in the levels of chemokines (IL-8 and MCP-1) and acute response cytokines (IL-1β and TNF-α). This, thus, facilitates a sustained increase in the proinflammatory cytokine IL-6 [46,69]. The excessive production of proinflammatory cytokines results in the destruction of immune system cells that exist to stop the SARS-CoV-2 infection. Therefore, the enhancing of the cytokine storm and the promotion of the damage and death of healthy cells, endothelial dysfunction, vascular damage, and paracrine/metabolic dysregulation in different organs [46,69] is induced. It has been demonstrated that the rain of cytokines promotes the development of several serious pathologies that are associated with COVID-19, including: SARS, pneumonia, septic shock, and metabolic acidosis. This is in addition to thromboembolic diseases, such as disseminated intravascular coagulation (DIC) or pulmonary intravascular coagulopathy (PIC) (which is caused by the occlusion of large vessels), acute ischemic cerebrovascular accidents, encephalitis, and acute kidney disease. These all cause multi-organ dysfunction syndrome, thereby worsening the prognosis and increasing the mortality rate [46,69].

The anti-SARS-CoV-2 pharmacological strategy (antivirals, antiretrovirals, or antimalarials) as a monotherapy is not sufficient in order to reverse the cytokine storm. Therefore, a combined anti-inflammatory and antiviral treatment is used [69]. The most common and effective anti-inflammatory drugs against this condition are immunomodulators such as tocilizumab, which is a monoclonal antibody that interacts with the IL-6 cell receptor and inhibits its proinflammatory effects. This is because other anti-inflammatory drugs, such as corticosteroids and nonsteroidal anti-inflammatory drugs (NSAIDs), have been associated with a higher mortality, increased risk of secondary bacterial or fungal infections, and prolonged stay in the intensive care unit [69]. However, the treatment of this hyperinflammatory response will depend on the patient’s immune profile, comorbidity, and polypharmacy [69]. In this context, the use of anti-inflammatory strategies of natural origin could have a positive impact in the treatment of this condition [37].

Multiple research articles and reviews have described saponins and sapogenins as important anti-inflammatory compounds capable of regulating the expression of various cytokines in different inflammatory models. The types that have been studied the most are the anti-inflammatory activity in the saponins of the species *Panax ginseng* [46,48]; *Bupleurum chinense* and *B. scorzonerifolium* [65,70]; *Glycyrrhiza glabra* [42]; and *Aesculus hippocastanum* [43,71]. However, due to the fact that, in severe COVID-19 patients, the hyperinflammatory condition of cytokine storm begins in the lungs (and then spreads to different organs [69]), those studies that have focused on and described the anti-inflammatory capacity of saponins within in vivo models or clinical studies of lung inflammation, takes major relevance [42,46,48,70,72].

A wide variety of triterpenoid saponins have been shown to exert anti-inflammatory effects within the in vivo models of pulmonary inflammation, including induced acute lung injury, ischemia–reperfusion lung injury, pulmonary fibrosis, sepsis, viral infection, and asthma [42,46,48,70,72]. Among them, *Panax notoginseng* saponins have been shown to inhibit the expression and secretion of proinflammatory cytokines (TNF-α, IL-1β, IL-6, and IL-8) and increase the expression of anti-inflammatory cytokines (transforming growth factor beta1 (TGF-β1) and IL-10) within in vivo models of lung inflammation, both in murine models [73] and in rabbits [74]. The in vivo modulation of proinflammatory and anti-inflammatory cytokines in lung tissue has also been demonstrated with the majority saponins of *P. notoginseng*. This has been achieved either by reducing oxidative stress via increasing the content of reduced glutathione (GSH) and by modulating the activity of superoxide dismutase (SOD), catalase (CAT), and myeloperoxidase (MPO) in treatments with Ginsenoside Rg3 [75]. It can also be achieved by decreasing the oxidative stress via increasing the expression of sirtuin 1 (SIRT1) in the endoplasmic reticulum in treatment with Ginsenoside Rg1 [76]. It could also be achieved by decreasing oxidative stress by inhibiting proinflammatory signaling pathways, including nuclear factor-kappa B(NF-kB) and mitogen-activated protein kinase (MAPK) pathways mediated by Toll-like receptor (TLR)-2 in Ginsenoside treatment Rb1 [77] and Ginsenoside Rg1 [78]. Another way could be in inhibiting the phosphatidylinositol 3 kinase (PI3K)/protein kinase B (Akt)/mammalian target of the rapamycin (mTOR) pathway, which is dependent on Mer receptor tyrosine kinase (MerTK) activation in treatment with Ginsenoside Rg3 [79], inhibiting the signaling pathway of TLR4 in treatments with Ginsenoside Rg5 [80], Ginsenoside Rh2 [81], and Ginsenoside Ro [82]. Additionally, both the isolated extracts of Radix Bupleuri and its isolated Saikosaponins have been shown to decrease the expression of proinflammatory cytokines (macrophage inflammatory protein-2 (MIP-2), IL-6, and TNF-α) and increase anti-inflammatory mediators (TGF-β1 and IL-10) within in vivo models of pulmonary inflammation [70]. Saikosaponin D was shown to modulate proinflammatory cytokines and to be an anti-inflammatory mediator via its reducing of oxidative stress, the rate of apoptosis in the lung, the concentration of MPO, and the infiltration of pulmonary neutrophils [83]. Furthermore, Saikosaponin-A has been shown to decrease the abnormal production of proinflammatory cytokines by its inhibiting of the NF-κB signaling pathway, NLRP3 inflammasome expression, and selective attenuation of lung neutrophil and monocyte recruitment [53,84]. In the same way, GLR has also been shown to inhibit pulmonary inflammation in vivo by decreasing the recruitment, migration, and infiltration of inflammatory cells. This is in addition to its collagen level; MIP-2; IL-4; IL-6; Granulocyte Macrophage Colony-Stimulating Factor (GM-CSF) and Interferon gamma (IFN-γ); its inhibiting of the expression of cyclooxygenase 2 (COX-2); inducible iNOS and NF-κB [42]; by its inactivation of the signaling pathway and expression of TLR-2 [85] and TLR4 [86,87]; and the activation of autophagy related to its downregulation of the PI3K/Akt/mTOR pathway [88]. Other triterpenoid saponins have also been shown to inhibit the expression of proinflammatory cytokines within in vivo models of pulmonary inflammation, either by inhibiting NF-κB and MAPK signaling pathways in treatments with Esculentoside A (isolated from *Phytolacca esculenta*) [89], with Anemoside B4 (isolated from *Pulsatilla chinensis*) [90], Astragaloside IV (isolated from *Astragalus membranaceus*) [91], Asiaticoside (isolated from *Centella asiatica*) [92,93] and with Tenuigenin (Isolate Polygala tenuifolia) [94]; by suppressing neutrophil infiltration and accelerating neutrophil clearance in treatments with Pseudoginsenoside-F11 (isolated from *Panax pseudoginseng* subsp. *himalaicus*) [95]; and by the downregulation of proapoptotic proteins (Caspase-3 and Bax), upregulation of the LXRα-ABCA1 pathway, and reduction in TLR4 translocation in treatments with Platycodin D (isolated from *Platycodon grandiflorum*) [96,97].

Recently, Escin has been proposed as a possible traditional medicine against severe acute lung injury related to COVID-19 infection, due to the fact that multiple clinical trials have demonstrated its potent anti-inflammatory and anti-edematous effects [43]. Escin has been shown to be a potent anti-inflammatory agent within in vivo models of pulmonary inflammation by its decreasing of the levels of proinflammatory cytokines (TNF-α, IL-1β, and IL-6), either through increased expression of glucocorticoid receptors and the endogenous antioxidant capacity [98], or the decrease in the expression of inflammasomes and pyroptosis through the downregulation of the signaling cascades of the high mobility group box 1 protein (HMGB1), TLR4, Myeloid differentiation primary response 88 (MYD88), MAPK, and NF-κB-p65 [99]. Currently, there are some clinical trials in progress with Escin or sodium escinate (injectable or in tablets) as an anti-inflammatory treatment in COVID-19 patients. Among them, a clinical trial (ChiCTR2000029742) carried out at the Tongji Hospital (Tongji Medical College, Huazhong University of Science and Technology, Wuhan, China) that proposed a randomized controlled trial, in parallel, in order to evaluate the efficacy and safety of the injectable sodium escinate when compared to conventional treatment in 90 patients with COVID-19 pneumonia. In addition, they compared the efficacy of conventional treatment plus injectable sodium escinate versus conventional treatment plus glucocorticoids. The anti-inflammatory efficacy of sodium escinate on patients with COVID-19 pneumonia is to be observed by chest imaging (computed tomography), C-reactive protein, and plasma IL-6 levels. Additionally, at the University of Catanzaro (Catanzaro, Calabria, Italy) a randomized, double-blind, parallel-controlled clinical trial (NCT04322344) is being carried out on 120 COVID-19 patients, which aims to evaluate the efficacy and safety of Escin as adjunctive treatment in patients infected with COVID-19, either as an oral formulation (40 mg 3 times a day) or injection (20 mg intravenously once a day) for 12 days. Further, a comparison with standard treatment will be made. In this clinical trial, the anti-inflammatory activity of Escin will be assessed by monitoring computed tomography scans and C-reactive protein.

**Figure 4:**
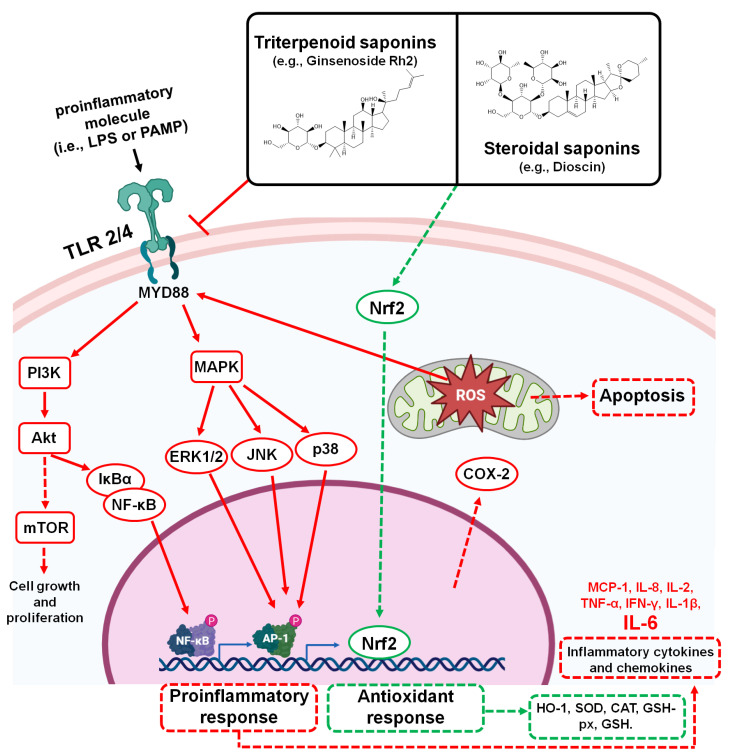
Main anti-inflammatory mechanisms of action of saponins in models of pulmonary inflammation. Both triterpene (e.g., Ginsenoside Rh2) and steroidal saponins (e.g., Dioscin) have been shown to reduce histopathological changes associated with inflammation in lung tissue by inhibiting the expression (mRNA and protein) and secretion of proinflammatory cytokines (TNF-α, TGF-β1R, IL-1b, IL-6 and IL-8) and increased expression of anti-inflammatory cytokines (TGF-β1 and IL-10). According to Passos et al. [26], Hsieh et al., [81], and Wang, et al., [104] the anti-inflammatory molecular mechanism of saponins commonly described for pulmonary inflammatory models involves the inactivation of the TLR2 and TLR4 signaling and expression pathway, since either inhibition of the TLR4/MYD88/PI3K/Akt signaling pathway or the TLR2/MYD88/MAPK pathway, thereby inhibiting the activation of NF-κB and AP-1, and consequently inhibiting gene expression associated with proinflammatory (proinflammatory enzymes and cytokines) and apoptotic responses. On the other hand, triterpene and steroidal saponins positively regulate the Nrf2 signaling pathways, promoting the expression of antioxidant genes, causing the expression of antioxidant enzymes and molecules (HO-1, SOD, CAT, GSH-px, GSH), decreasing cell death associated with oxidative stress by the inflammatory process. Red boxes and arrows indicate inhibition; Green boxes and arrows indicate stimulation.

On the other hand, it has been demonstrated that the anti-inflammatory effects of steroidal saponins within in vivo models of inflammation are exerted by acting directly on proinflammatory cytokines, inhibiting the action of macrophages, regulating the arachidonic acid pathway, and reducing the activity of COX-2 and prostaglandin E2 [26]. In in vivo models of lung inflammation, most studies have been conducted with Dioscin, a steroidal saponin (isolated from the *Dioscorea* species) that has been shown to reduce the secretion of proinflammatory and profibrotic cytokines (TNF-α, TGF-β, IFN- γ, IL-1β, IL-2, IL-4, IL-6, IL-13, and IL-17A). It has also been shown to modulate innate and adaptive immune responses (which includes the inhibiting of the activation and infiltration of fibroblasts, macrophages, and lymphocytes [26]). This is achieved through different mechanisms of action, including with the inhibition of apoptotic signal-regulating kinase 1 (ASK1)-p38/c-Jun N-terminal kinase (JNK) signaling [100], the inhibition of the NF-κB signaling pathway and the activity of the enzymes COX-2 and MPO [101]; the inhibition of the TLR4/MyD88 signaling pathway [102,103,104]; the increasing of the expression of α-glucocorticoid receptors (SLPI, GILZ, and MKP-1); the inhibition of the expression of the 70 kilodalton heat shock proteins (HSP70) [105]; and the promotion of alveolar macrophage autophagy through downregulation of the Akt/mTOR pathway [106]. Other steroidal saponins such as Trillin (isolated from *Dioscorea nipponica*) [107] and Timosaponin A-III (isolated from *Anemarrhena asphodeloides*) [108] have also been shown to be active within in vivo models of lung inflammation by the decreasing levels of TNF-α, IL-1β, and IL-6 through the inhibition of NF-κB and MAPK signaling pathways. Table S1 summarizes the main studies related to in vivo anti-inflammatory effects of saponins in models of pulmonary inflammation. The main anti-inflammatory mechanisms of action of saponins in models of pulmonary inflammation are provided in Figure 4.

#### 3.2.2. Antiplatelet–Antithrombotic Activity of Saponins and Their Potential Effects against Pulmonary Coagulopathies

It has been described that, as race and ethnic origin can have important effects on thrombotic risks, in Caucasian patients severe SARS-CoV-2 infections have been significantly associated with the development of different cardio/pulmonary coagulopathies that increase disease severity, thereby suggesting racial susceptibility to COVID-19 mortality [67]. Among the thrombotic events that occur in a late stage of a COVID-type disease is the disseminated intravascular coagulation (DIC) characterized by a marked increase in D-dimer levels, as well as a consistent progressive activation of coagulation, which generates venous thromboembolism [67,68]. Additionally, the diffuse bilateral pulmonary inflammation observed in COVID-19 patients has been described to be associated with a new specific pulmonary thromboembolism called “pulmonary intravascular coagulopathy (PIC)” [67]. It has been argued that the biological mechanisms underlying these pulmonary vasculopathies in severe COVID-19 patients are related to the human ACE2 receptor used by SARS-CoV-2 to bind to and enter the host cell. This is because it is expressed both in type II pneumocytes as well as in vascular endothelial cells within the lungs, raising the possibility that the pathobiology of SARS-CoV-2 may include infection, activation, or the direct lung damage of endothelial cells and thus causing pulmonary coagulopathies [67,68]. It has been demonstrated that the initial anticoagulant treatment of COVID-19 patients with associated coagulopathies should be with low molecular weight heparin (LMWH). This is because it can reduce mortality (up to 48% at 7 days and 37% at 28 days) and also because it manages to significantly improve arterial oxygen pressure/inspired fraction of O_2_ (PaO_2_/FiO_2_). This prevents the formation of microthrombi and associated pulmonary coagulopathies, as well as reduces complementary inflammation [68]. In this context, multiple studies have described saponins as modulators of the blood coagulation system, proposing these compounds for preventive treatment and complementary treatment to low molecular weight anticoagulants in different thromboembolic diseases [21,47,109,110,111,112,113]. In fact, the antiplatelet/antithrombotic effects of saponins have been studied in both in vitro and in vivo models, demonstrating different mechanisms of action depending on their structure [21]. Among them, it has been described that the inhibitory effects of saponins on platelet activation are related to the increase of cyclic adenosine monophosphate (cAMP) and cyclic guanosine monophosphate (cGMP), mobilization and reduction in the Ca^2+^ level, and the inhibition of granule secretion with platelet agonists [21,47]. Additionally, their antithrombotic effects have been associated with their ability to inhibit platelet aggregation by the inhibiting of different coagulation factors, including factor VIII [21,47]. However, despite the antiplatelet/antithrombotic potential of saponins, there are few studies that link their use in models of pulmonary thromboembolism or in other coagulopathies that are associated with COVID-19 patients.

Li et al. [109] evaluated the antithrombotic activity of total steroidal saponin (TSS) extracts from *Dioscorea zingiberensis* (DZW) rhizomes, whose most abundant saponins were protodeltonin (14–26%), deltonin (4–12%), parvifloside, (5–11%) and zingiberensis saponin (3–8%). The antithrombotic effect of TSS-DZW was evaluated ex vivo by measuring adenosine 5’-diphosphate (ADP)-induced platelet aggregation in rats while they were given a daily oral treatment of TSS-DZW (32.3, 64.7, and 129.4 mg/kg for 2 weeks). In addition, there was a measuring, in vivo, of the thrombus growth inhibition rate (weight) in a rat model of inferior vena cava ligation thrombosis. Additionally, and as a complement, plasma coagulation parameters and the rate of protection against death were also measured in mice. These mice had TSS-DZW orally administered to them daily (45.3, 90.6, and 181.1 mg/kg for 2 weeks) before the induction of pulmonary thromboembolism (which was applied via the injection of collagen and epinephrine). Results showed that TSS-DZW significantly inhibited (25% inhibition at 129.4 mg/kg dose) ex vivo ADP-induced platelet aggregation and reduced the size of inferior vena cava ligation-induced thrombus in rats by up to 84%. TSS-DZW significantly prolonged the time of coagulation parameters (activated partial thromboplastin (APTT), thrombin time (TT), and prothrombin time (PT)) and bleeding time in mice, in a dose-dependent manner. Similarly, TSS-DZW provided significant protection of up to 45.4% against death from pulmonary thrombosis in mice. It was argued that the effect of TSS-DZW was due to the inhibition of thrombosis that depended mainly on the inhibition of platelet aggregation, suggesting its major saponins (protodeltonin, deltonin, parvifloside, and zingiberensis saponin) as potential candidates that could reduce the risk of thrombotic cardio/pulmonary diseases.

Chen et al. [110] evaluated the antithrombotic effect of Naodesheng (NDS), which is a traditional Chinese medicine that is widely used for the treatment of cerebral arteriosclerosis, ischemic stroke, cerebral hemorrhage sequelae. Further, NDS also contains an abundant content of saponins, such as Notoginsenoside R1 and Ginsenoside (Re, Rg1, Rb1, Rb2, Rb3, Rc, and Rd) due to the fact that it is formulated by Chuanxiong Rhizoma, Lobed Kudzuvine, *Carthamus tinctorius*, Radix Notoginseng, and the *Crataegus pinnatifida* species. In this study, a bioactive fraction of NDS (CD hydroalcoholic fraction) was used, which had a total content of 7.9% saponins. The antithrombotic effect of the CD fraction of NDS was evaluated ex vivo by measuring the platelet aggregation that was induced by ADP in rats, to which a daily dose of 1.01 g of CD/kg weight was administered by gavage for 5 days. Furthermore, there was a measuring of the coagulation time, in vivo, as well as a measuring of the protection rate in mice to which a daily dose of 2.14 g of CD/kg weight was administered by tube for 5 days, prior to the induction of pulmonary thromboembolism (via the injection of collagen and epinephrine). The results showed that the CD fraction significantly inhibited (22.48%) platelet aggregation in rats, and that it prolonged the coagulation time to 108.3 s in mice. Therefore, the obtained results were comparable to the reference compound (Aspirin: 23.45% inhibition and 112.4 s in the clotting time, respectively). Similarly, in mice with pulmonary thromboembolism, a 60% protection from death or paralysis was reported (Aspirin 70% protection). The authors indicated that the antithrombotic effect of the CD fraction of NDS was due to its ability to inhibit platelet aggregation.

Zhang et al. [101] evaluated the antithrombotic effect of diosgenin, a steroidal saponin extracted from the rhizome of *Dioscorea zingiberensis*, as well as of four derivatives of it (synthetic and natural) (i.e., diosgenyl saponins). Preliminarily speaking, it was reported that the compound diosgenyl β-D-galactopyranosyl-(1→4)-β-D-glucopyranoside (C3)—a derivative of diosgenin with a disaccharide attached at the C-3 position—was the compound that most prolonged bleeding time (652.5 s) in a dose-dependent manner in the mice that were orally pretreated with C3 (25–100 µM), twice a day for 5 days. Additionally, C3 demonstrated a dose-dependent inhibition of platelet aggregation induced by ADP and thrombin. Significant results of up to 15% inhibition in rats pretreated with C3 (100 mg/kg), when compared to the untreated control, were observed. In the mice that were pretreated with C3 at a dose of 100 mg/kg, the APTT was significantly prolonged (20.95 s) compared to the control group (16.73 s). Moreover, they significantly and dose-dependently inhibited the activities of factor VIII up to 35% (100 mg/kg), but no effect was observed on the activities of other factors (FIX, FXI, and FXII). On the other hand, dose-dependent protection against death by pulmonary thrombosis (induced by injection of collagen and epinephrine) was reported in mice, with a significant protection of up to 45.45% being observed in the group pretreated with 100 mg/kg when compared to the group treated with 100 mg/kg (i.e., the control group (8.33%)). Based on these results, the authors suggest that the inhibition of factor VIII activities and platelet aggregation are the mechanisms of action by which C3 inhibits pulmonary thrombosis in mice.

Shen et al. [112] evaluated the antiplatelet effect of saponins from *Panax notoginseng* (PNS), a traditional Chinese medicine widely used to treat blood coagulation, inflammation, and pain. The study used a commercial PNS lyophilizate (purity > 99%) rich in Ginsenoside Rg1, Ginsenoside Rb1, and Notoginsenoside R1. They then evaluated its in vitro effect at concentrations of 1, 10, 100 μg/mL on thrombin-induced platelet aggregation and its possible molecular mechanism of action. In addition, in vivo antithrombotic activity was evaluated by coagulation indicators such as APTT, PT, and fibrinogen in a hypercoagulable rat model, to which PNS was orally administered (10, 100 and 200 mg/kg) one hour before thrombin infusion (i.e., the hypercoagulable state). The results of this study demonstrated that PNS treatment inhibited platelet aggregation by up to 20% when utilizing 100 μg/mL treatments. This inhibition of platelet aggregation was associated with up to a 2-fold overexpression (mRNA and protein levels) of the peroxisome proliferator-activated receptor gamma (PPAR-γ), which is a recently described nuclear receptor on platelets that, when is activated, could inhibit platelet activation and aggregation by the downregulation of signaling the downstream of the GPVI collagen receptor. A positive regulation of PPAR-γ on the PI3K/Akt/iNOS pathway was also demonstrated; further, it was argued that the activation of this pathway would result in the synthesis of platelet nitric oxide (NO), the elevation of cGMP, and the inhibition of the secretion of granules with platelet agonists, thereby promoting the inhibition of platelet activation and aggregation. In addition, PNS was shown to significantly reverse the hypercoagulable state induced in rats. This is because it prolonged APTT and PT coagulability parameters to values similar to the normal control, as well as also decreased fibrinogen expression. The authors concluded that the antiplatelet mechanisms of PNS are mainly related to the activation of the PI3K/Akt/iNOS pathway in a PPAR-γ-dependent manner. Due to this, they propose its potential use in thromboembolic diseases, such as pulmonary embolism.

**Figure 5:**
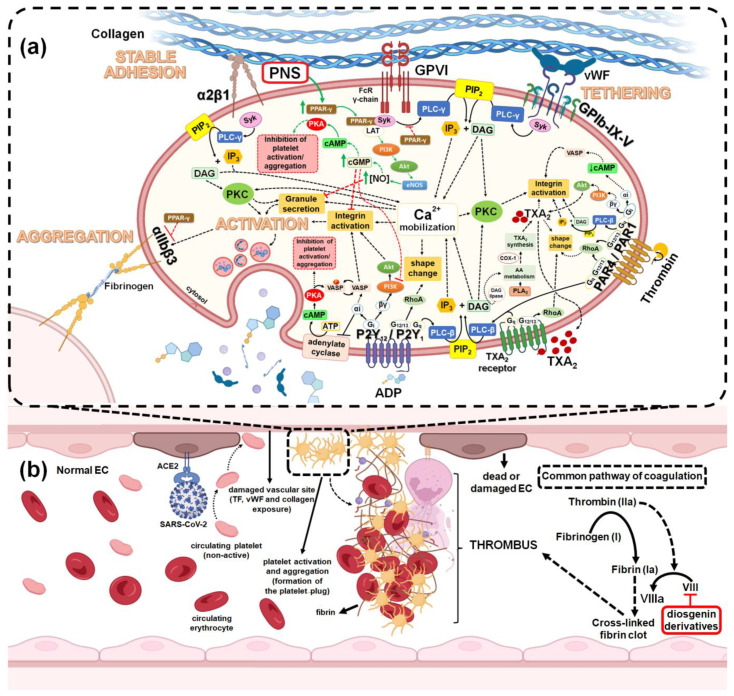
Main antiplatelet-antithrombotic mechanisms of action of saponins in models of pulmonary coagulopathies. (**a**) Antiplatelet mechanism of saponins. According to Olas et al., [21], Lee et al., [47], and Gómez-Mesa et al., [114], after damage and death of vascular endothelial cells (EC) occurs due to the SARS-CoV-2 infection, the circulating (non-active) platelet recognizes the exposure of components of the subendothelium (TF, vWF and collagen) at the damaged vascular site, promoting different events, including: the initial “anchoring or tethering” of the platelet, characterized by transient interactions between vWF immobilized in the subendothelial matrix and to the GP Ib-IX-V glycoprotein complex present on the platelet surface. This union between “agonists” and their respective platelet receptors triggers different signaling pathways that culminate in “platelet activation and aggregation”, an event that promotes the formation of the “platelet plug”. Platelet activation mediated by “extracellular matrix agonists” (such as vWF and collagen) involves the participation of different glycoproteins (GP Ib-IX-V, GPVI and α2β1, respectively) and their signaling pathway is characterized by the recruitment and activation of Syk, which promotes the activation of the enzyme phospholipase c (PLC)-γ that hydrolyzes membrane phospholipids (Phosphatidylinositol 4,5-bisphosphate, PIP2), producing the mediators diacylglycerol (DAG) and inositol 1,4,5-trisphosphate (IP3) that promote the mobilization of intracellular Ca^2+^. The increase in intracellular Ca^2+^ induces the activation of signaling pathways that converge in the activation of the αIIbβ3 integrin, and also promotes changes at the cytoskeleton level, which converge in changes in platelet shape, such as the extension of filopodia and lamellipodia until the complete spreading or full cell spreadin. DAG can be sequentially hydrolyzed by diglyceride lipase and promote the formation of arachidonic acid for the synthesis of Thromboxane A2 (TXA_2_). Furthermore, the increase in DAG and Ca^2+^ promote the activation of the protein kinase C (PKC) enzyme, which participates in different signaling pathways, among them it initiates the αIIbβ3 integrin activation cascade to produce platelet aggregation, and also activates signaling pathways that cause the mobilization and secretion of granules (dense and alpha) that contain “soluble agonists” that stimulate and promotes the activation of other platelets. Platelet activation mediated by “soluble agonists” (such as ADP, thrombin and TXA_2_) involves the participation of different G protein-coupled receptors, which ensure and promotes the amplification of the platelet activation and aggregation response through different signaling pathways. ADP acts as an agonist at P2Y_1_ (coupled to G_q_ and G_12/13_) and P2Y_12_ (coupled to G_i_) receptors. Thrombin acts as an agonist of receptors activated by proteases (PAR) of the PAR1 (coupled to G_q_, G_12/13_ and G_i_) and PAR4 (coupled to G_q_ and G_12/13_) type. TXA_2_ acts as an agonist of the TXA_2_ receptor (coupled to G_q_ and G_12/13_). G_q_-associated signal transduction is characterized by regulation of intracellular Ca^2+^ levels and activation of PKC through the PLC-β→IP3/DAG pathway to promote shape change, integrin activation, granule secretion and TXA_2_ synthesis. G_12/13_-associated signal transduction is characterized by activation of the GTPase Rho isoform A (RhoA), which promotes platelet shape change by regulating actin cytoskeletal dynamics. G_i_-associated signal transduction is characterized by the action of its two subunits, which through different pathways promote the stabilization of platelet aggregation through the activation of the αIIbβ3 integrin. The αi subunit promotes the inhibition of adenylate cyclase and the consequent inhibition of VASP phosphorylation. The βγ subunit promotes the PI3K/Akt signaling pathway. Most studies related to models of pulmonary coagulopathies have been carried out with the main saponins of Panax notoginseng (PNS) (e.i. ginsenoside Rg1, ginsenoside Rb1 and notoginsenoside R1) (red box) [110,112,113]. PNS inhibit collagen-induced platelet activation by promoting a downregulation of signaling downstream of the GPVI receptor. This occurs through an overexpression of PPAR-γ, and upregulation of the PPAR-γ-dependent PI3K/Akt/eNOS pathway, which results in increased nitric oxide (NO) synthesis and consequent increase in cGMP levels, which prevents the activation of platelets through different mechanisms: the indirect increase in cAMP levels by inhibition of phosphodiesterase 3 (PDE-3); the increase in cAMP levels acts synergistically with that of cGMP to inhibit platelet aggregation; inhibits the activation of phosphatidylinositol-3 kinase (PI3K) which leads to the activation of integrin αIIbβ3; and produces phosphorylation of the TXA_2_ receptor and inhibits its function. In addition, and independently of cGMP production, NO inhibits exocytosis of platelet granules. (**b**) Mechanism of antithrombotic action of saponins. The antithrombotic activity of saponins on models of pulmonary coagulopathies derives from the ability to inhibit platelet aggregation and formation of the platelet plug (primary hemostasis) and, consequently, prevent the cross-linked fibrin clot and thrombus (secondary hemostasis). However, diosgenin derivatives also have the capacity to inhibit the factor VIII of the common pathway of the coagulation cascade [111]. Factor VIII is activated by thrombin (IIa) and promotes the formation of covalent bonds that cross-link the fibrin polymers (formed from activated monomers) leading to the thrombus formation. Inhibition of factor VIII by diosgenin derivatives (red box) prevents the common pathway of the coagulation cascade and fibrin thrombus formation.

Recently, Wang et al. [113] evaluated the antithrombotic potential of Xuesaitong, a Chinese patent medicine (Hunan Xiangya Pharmaceutical Co., Ltd., China; 1609101) that is mainly made up of PNS. The prospective cohort clinical study was carried out between January 2016 and November 2018 at Xuanwu Hospital (Capital Medical University, Beijing), and included 281 hospitalized surgical patients (>18 years) with moderate to high risk of deep vein thrombosis (DVT). The patients were divided into two groups: the control group (147 patients), who were treated with low molecular weight heparin (LMWH) via hypodermic injection (4000–8000 AxaIU, once daily); then there was the exposure group (134 patients) who were treated with PNS by oral Xuesaitong tablets (100 mg, 3 times a day), combined with the LMWH that was also administered to the control group. The antithrombotic effect of PNS was assessed by diagnosing DVT, thromboelastography (TEG) values, and by measuring coagulation function tests, including PT, APTT, TT, fibrinogen, international normalized ratio (INR), and D-dimer. The monitoring of the parameters was carried out during the hospital stay of all patients. Among the most relevant results it was observed that there was a significant decrease in the incidence of DVT in the exposure group (PNS + LMWH) with 21 incidents (15.7%) of DVT, compared to the control group (LMWH), which had 41 incidents (27.9%) of DVT. In both groups, the participants diagnosed with DVT were older and had a high level of D-dimer. No significant changes were observed between the groups in TEG values and coagulation function tests. The authors argued that this antithrombotic effect of the PNS + LMWH treatment was due to the complement of bioactive saponins such as Notoginsenoside R1, Ginsenoside Rg1, and Ginsenoside Rb1, which are characterized by their anti-inflammatory, antiplatelet, and antithrombotic activity, among others. Finally, based on the results, it was concluded that the combination of PNS and LMWH significantly reduced the incidence of DVT without increasing the risk of bleeding when compared to LMWH treatment alone. In addition, it was postulated as a potential adjunctive treatment in surgical patients with the risk of DVT and who are treated with low molecular weight anticoagulants due to serious complications, such as symptomatic pulmonary embolism. Table S2 shows a summary of the main studies related to the antiplatelet-antithrombotic effects of saponins in clinical trials and in vivo models of pulmonary coagulopathies. The main antiplatelet-antithrombotic mechanisms of action of saponins in models of pulmonary coagulopathies are provided in Figure 5.

### 3.3. Immunostimulatory Activity of Saponins and Their Potential Uses as Adjuvants for the Development of Anti-SARS-CoV-2 Vaccines

Currently, population vaccination has been implemented as part of the global strategy in order to reduce infections and to mitigate the effects of the coronavirus disease. This approach is considered a safe and effective method in order to provide protection and to reduce the spread of the virus [115,116,117]. In this sense, more than 300 candidate vaccines against SARS-CoV-2 have been proposed in different stages of development, of which more than 100 have reached clinical trials [115,116]. The European Medicines Agency (EMA) has authorized six vaccines against COVID-19 for use in the European Union, including COVID-19-VLA2001 (Valneva), Vaxzevria (AstraZeneca), Spikevax (Moderna), Comirnaty (Pfizer/BioNTec), Janssen (Johnson & Johnson), and Nuvaxovid (Novavax) [118]. However, only four of these vaccines were emergency use authorized (EUA) by the Food and Drug Administration (FDA) (Spikeva, Comirnaty, Janssen, and Nuvaxovid) [119]. These vaccines have shown different degrees of effectiveness (between 52.9% and 100%), which will depend on the type of vaccine (mRNA vaccines: Comirnaty and Spikevax; those based on adenovirus vectors: Janssen and Vaxzevria; inactivated virus: COVID-19-VLA2001; and vaccines with antigenic subunits: Nuvaxovid (recombinant S protein)) and adjuvants that improve or modulate their specific immune response [116]. Despite vaccination campaigns, the appearance of different variants of SARS-CoV-2 has caused the reappearance of recent pandemic waves, causing devastation worldwide and concerns about the efficacy of drugs and vaccines, which are still under investigation [116,117]. As a result, there is currently a therapeutic need to improve the efficacy and safety of vaccines against COVID-19. This has, in turn, reinforced the importance of innovation and the use of better immunostimulants for the production of vaccines that provide better, safe, and long-term immunological protection [49,116,117,120]. In this context, it has been demonstrated that the protective immunity of certain types of vaccines could be enhanced by the addition or substitution of different adjuvants [120,121]. Between these adjuvants, saponins are one of the most widely used immunostimulants to boost vaccines because they may modify the activities of T cells and activate anti-gen-presenting cells (APCs), such as dendritic cells (DC), and induce Th1/Th2 adaptive immunoresponses (Th1 or cell-mediated immunity controlled by activated T cells, and Th2 or humoral immunity controlled by activated B cells and antibodies) or only Th2 response [36,49,120,121,122,123,124,125].

Among the saponins with immunostimulatory activity, those obtained from the bark of the Chilean quillay tree or *Quillaja saponaria* Molina (QS), are the most studied and widely used as adjuvants. This is because they can be used to improve humoral and cellular immune responses to vaccines [124]. For example, different natural QS with immunostimulatory activity have been purified and identified by reverse-phase high-performance liquid chromatography (RP-HPLC), including QS-7, QS-17, QS-18, and QS-21 (predominant saponins in the bark) [121,125]. These have been used in vaccines, either in the form of free adjuvant (e.g., QS-21) in formulations with other immunostimulants (e.g., AS01, AS02, and AS15), in immunostimulant complexes (e.g., ISCOM) or in structures (e.g., Matrix-M1) [121,126]. QS fraction 21 (QS-21), an acylated 3,28-bisdesmodic triterpene glycosides (1,3) is the fraction with the highest immunostimulatory activity [125]. This has been used as the key component in various (licensed) adjuvant systems that have been selected for the development of different efficient and safe vaccines (and approved by the FDA and EMA). Table 2 shows the (licensed) saponin-based adjuvant systems that have been used in the development of vaccines approved for human use.

**Figure 6:**
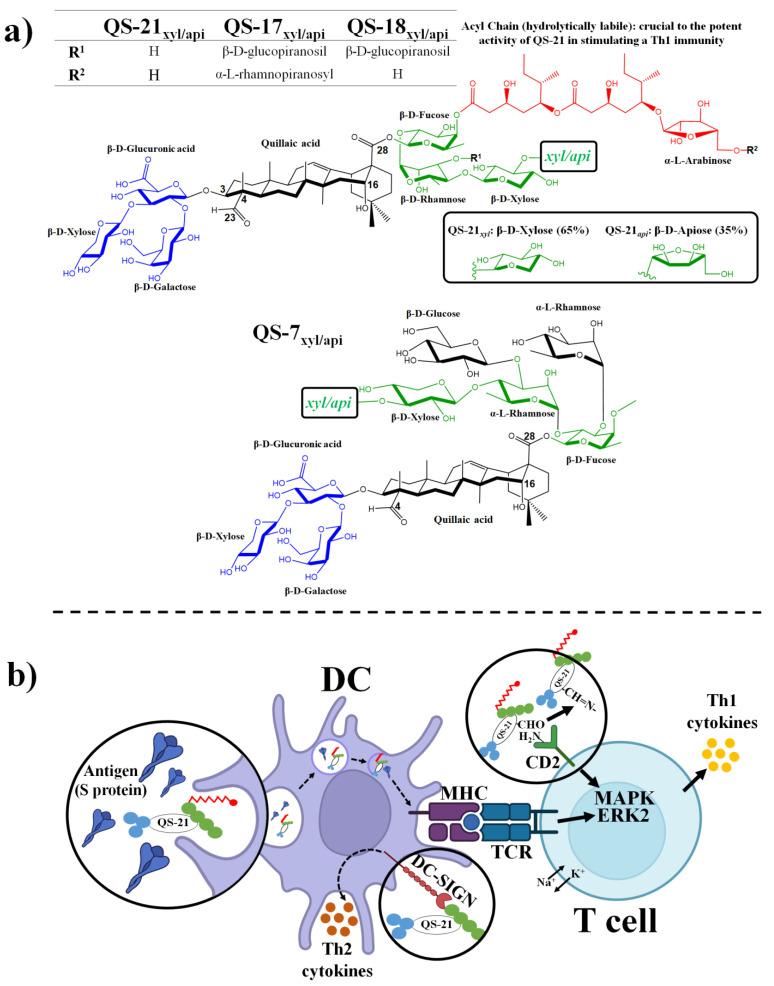
Structure-activity relationship of saponins from *Quillaja saponaria* and adjuvant mechanism of action of QS-21. According to Wang et al. [121] and Marciani et al. [123], QS-21 is characterized by having a triterpene aglycone nucleus, quillaic acid (black), which contains an aldehyde group attached to C4 (carbonyl C23), a hydroxyl group attached to C16, and a carboxyl group at C28. The quillaic acid core is substituted with a branched trisaccharide (blue: β-D-GlcA, β-D-Xyl β-D-Gal) that is connected via a beta-glucosidic ether bond to the hydroxyl group at C3. It also has a reducing end composed of linear tetrasaccharide (green: β-D-Fuc, β-D-Rha, β-D-Xyl, β-D-Xyl/β-D-Api) beginning with a fucose residue attached by a beta glycosidic ether bond at C28. QS-21 (as well as QS-7, QS-17, and QS-18) represents a mixture of the xylose and apiose substituted variants (65:35). The fucopyranosyl residue at position C28 is in turn connected to an acyl chain with a terminal arabinose (red) through a hydrolytically labile ester. The main structural difference between QS-21 and QS-17/18 is in the C28 oligosaccharide domain. Instead of having a linear tetrasaccharide as in QS-21, QS-17/18 has an additional β-D-glucopyranosyl unit (R^1^ group) connected to the α-L-rhamnopyranosyl unit at its 3-O position. QS-17 differs from QS-18 only in the R^2^ group of the acyl side chain, i.e., QS-17 has a disaccharide unit while QS-18 has a monosaccharide unit at the other end of the side chain. On the other hand, QS-7 is characterized by not having an acyl chain in the C28 oligosaccharide domain and the latter with 2 more sugar residues than QS-21 (one β-D-glucopyranosyl unit in position R1 and α unit -L-rhamnopyranosyl connected in position 3-O of the fucose residue). Although QS have a similar adjuvant activity profile (can stimulate a Th1/Th2 response), they have different toxicity profiles (QS-17/18 > QS-21 > QS-7) [121,123]. However, the immunostimulatory activity and toxicity of QS are probably determined by the specific molecular structure of each individual saponin, rather than by the presence/absence of a certain structural feature in the saponin or by its amphipathicity [121,123,130]. On the other hand, considering that a Th1 response is always followed by a Th2 type, and that Th2 can exist as a single type of immunity [123], the structure-activity relationship of QS-21 has shown that acyl chain removal it leads to loss of the ability to stimulate a Th1 response (production of cytotoxic T lymphocyte (CTL)) [121,123,130]. However, when the loss of the acyl chain occurs, the fucosyl unit at the reducing end of the C28 oligosaccharide is exposed for its interaction with C-type lectin receptors (CLRs), specifically, with dendritic cell-specific intercellular adhesion molecule3-grabbing non-integrin (DC-SIGN) receptors, skewing the DCs to a single Th2 immunity [121,123,133]. It has also been described that the aldehyde group in position C4 of the saponin can form an imine with the ɛ-amino group of the T lymphocyte receptor (most likely CD2) triggering the activation of the MAPK signaling pathway, changes in transport channels of K^+^ and Na^+^, and finally skewing the activated T cells to a Th1 immunity with increased production of Th1 cytokines [49,122,124]. (b) Immunostimulatory mechanism of action for QS-21. According to Zhang et al., [138], the mechanism of action for QS-21-based adjuvants (AS01, ISCOM, ISCOMATRIX, and Matrix M) is characterized by the following: DCs arrive at the site where the vaccine was inoculated, and through endocytosis, they incorporate into their interior the exogenous protein antigens (e.g., S protein from SARS-CoV-2) and QS-21. Following QS-21-mediated endosomal membrane disruption (attributed to acyl chain action), protein antigens are degraded by the proteasome into smaller peptide fragments. Protein fragments are transported to the ER by carrier molecules, where chaperones facilitate their binding to newly synthesized MHC-I molecules for vesicular migration through the Golgi apparatus to the cell surface. Finally, surface-exposed peptide epitopes of DC in association with MHC-I molecules are presented to naive CD8^+^ T cells (cross-presentation) via the T cell receptor (TCR) stimulating a Th1 response. QS-21 can act on both DCs and T cells. Oligosaccharide chains of saponins can activate DC by binding to surface-expressed DC-SING, skewing the DC to a Th2 response. The aldehyde group of quillaic acid forms an imine with an amino group of a T-cell surface receptor (CD2), which sends an intracellular signal that activates MAPK/ERK2 and with changes in cellular K^+^ and Na^+^ transport, skewing the T cells to a Th1 response.

In its natural form, QS-21 is a mixture of two structural isomers, both of which share a quillaic acid nucleus (with an aldehyde group at C4 and a carboxyl group at position C28), a branched trisaccharide connecting to the C3 OH group (via a beta glycosidic ether linkage), and a linear tetrasaccharide connecting to the C28 carboxyl group (via a beta glycosidic ester linkage). Furthermore, it is also where the reducing end of the C28 tetrasaccharide is a β-D-fucosyl unit with its 4-O position capped with a glycosylated pseudodimeric fatty acyl chain [127]. The difference between the QS-21 isomers lies in the non-reducing end of the C28 tetrasaccharide, which may have a terminal β-D-apiosyl unit (QS-21api with a 65% abundance) or a terminal β-D-xylosyl unit (QS-21xyl with 35% of abundance) (Figure 6) [127,128,129]. The mechanisms of the immunostimulatory action of QS-21 and the different (licensed) adjuvant systems, was extensively studied and discussed [49,121,123,127,128,129,130,131,132,133]. Recently, a dual action mechanism was proposed in QS-21-based adjuvant systems, where this saponin would act on both T cells and antigen-presenting cells (dendritic cells) in a receptor-mediated and non-receptor-mediated manner, respectively [123,133]. Specifically, it has been described that by intramuscularly injecting an antigen and the adjuvant AS01 (QS-21 + 3-O-desacyl-4′-monophosphoryl lipid A (MPL)), endocytosis of the complex occurs via the dendritic cells (DC), such that MPL then activates TLR4, and QS-21 activates the NLRP3 inflammasome. This, therefore, results in DC activation and the release of IL-1β and IL-18 [134,135]. MPL and QS-21 act synergistically in order to increase chemokine release, circulate granulocytes, and enhance monocyte and dendritic cell recruitment [131,136]. In addition, it promotes the mobilization and activation of immune cells in the draining lymph nodes, a place where highly activated dendritic cells effectively induce the differentiation of naïve CD4+ T cells into CD4+ memory T cells and CD4+ effector T cells. Cytokines secreted by CD4+ effector T cells (such as IL-2, TNF-a, CD40L, and IFN-c) could stimulate the division of naive B cells into plasma cells and memory B cells [123,133]. Additionally, the aldehyde group present in the C4 position of the sapogenin could react with the ε-amino groups. This would be achieved most likely due to the CD2 receptor on T cells, which would form an imine that provides the T cells with a signal (activation of the MAPK signaling pathway, which takes place together with changes in the cellular transport of K+ and Na+) that is necessary for T cell activation biased towards the Th1 response and the resulting secretion of Th1 cytokines [137,138]. Recently, a new mechanism of action for QS-21-related saponin adjuvants was discussed. In this, it was proposed that upon the endocytosis of antigens and QS-21 via dendritic cells, QS-21 causes a destabilization and rupture of the endosomal membrane (due to its amphiphilic properties), thereby causing the activation of dendritic cells, the production of proinflammatory cytokines, and the release of antigens to the cytosol. All of these are then processed by the machinery of the proteasome into smaller peptide fragments. They are then moved to the ER via carrier molecules, where chaperones facilitate their binding to newly synthesized major histocompatibility complex class I (MHC-I) molecules for the purposes of vesicular migration through the Golgi apparatus to the cell surface. Finally, the peptide epitopes of the antigen are exposed on the DC surface in association with MHC-I molecules and are presented to naive CD8+ T cells (cross-presentation) via the T cell receptor (TCR) [131,133]. The structure-activity relationship of saponins from *Quillaja saponaria* and the adjuvant mechanism of action of QS-21 are provided in Figure 6.

Due to its immunostimulatory mechanism of action, different QS-based adjuvant systems have been used in approved vaccines that were shown to be safe and effective against different intracellular pathogens, including the varicella herpes zoster virus vaccine (Shingrix^®^, approved by the FDA) [139], or against the malaria parasite Plasmodium falciparum (Mosquirix^®^, approved by the EMA) [131]. Recently, the Nuvaxovid™ (NVX-CoV2373) vaccine was approved (20 December 2021) within the USA by the World Health Organization (WHO) for the purposes of active immunization and the prevention of the COVID-19 disease caused by SARS-CoV-2 in people older than 12 years [116]. Nuvaxovid™ (also manufactured in India under the trade name Covovax™) is a recombinant nanoparticle vaccine of the S protein (viral glycoprotein that binds to the ACE2 receptor of the host cell) combined with the adjuvant Matrix-M (which is based on different immunostimulatory fractions of *Q. saponaria*). This vaccine elicits the immune responses of B lymphocytes and T lymphocytes to the viral glycoprotein S of SARS-CoV-2 [49,116]. Clinical trials with this Matrix-M-adjuvanted vaccine have shown an efficacy of 90.4–100% against moderate to severe diseases [116]. It has also been shown that SARS-CoV-2 variants, including the Ómicron variant (i.e., the most infectious), have multiple mutations in the RBD region of the S protein and, as a result, more than 85% of the neutralizing antibodies generated by vaccinated individuals have not been entirely effective, thereby posing a serious threat to existing therapies, including COVID-19 vaccines [140]. Against this, some authors have proposed the NVX-CoV2373 vaccine as an alternative to better deal with the evolved variants of SARS-CoV-2 [36,49]. This may be due to the fact that this Matrix-M-adjuvanted vaccine is composed of the complete S protein, which has different epitopes in common with the different mutations present in the SARS-CoV-2 variants, generating better titers of anti-S IgG antibodies and better neutralization responses against the different variants. However, more clinical studies are still needed to confirm this [36,49,116]. Table 2 shows the (licensed) saponin-based adjuvant systems that have been used in the development of vaccines approved for human use.

**Table 2:** Saponin-based adjuvant systems (licensed) that have been used in the development of approved vaccines for human uses.

Adjuvant System	Composition	Approved Vaccine	Activity	Refs.
AS01 (GlaxoSmithKline)	A combination of immunostimulants QS-21 and MPL with liposomes	Shingrix/GlaxoSmithKline (Anti-varicella-zoster virus, FDA approved). Mosquirix/GlaxoSmithKline (Anti-malaria, EMA approved)	Increases Th1 and Th2 responses. It is characterized by increasing the antigen-specific antibodies and the frequency of antigen-specific CD4^+^ T cells. CD4+ T cells express higher amounts of cytokines such as IL-12, TNF-α, and IFN-γ and promotes a higher specific memory B cell response.	[128,141]
AS02 (GlaxoSmithKline)	A combination of immunostimulants QS-21 and MPL with an oil in water emulsion	-	Increases Th1 and Th2 responses. It is characterized by increasing the antigen-specific antibodies and the frequency of antigen-specific CD4^+^ T cells. CD4+ T cells express higher amounts of cytokines such as IL-12, TNF-α, and IFN-γ and promotes a higher specific memory B cell response.	[128,142,143,144]
AS15 (GlaxoSmithKline)	A combination of immunostimulants QS-21, CpG 7909 and MPL with liposomes	-	Increases plasmacytoid DC and B cells through TLR-9 and TLR-4 potentiating the Th1 pathway with higher IFN-γ and IL-12 secretion by dendritic cells. In antitumor vaccines increases the average antibody titers against tumor-specific antigen, and the anti–tumor antigen CD4^+^ T cells.	[128,145,146]
ISCOM-Matrix (Novavax)	An antigen delivery system, cage-like self-assembled nanoparticles (40 nm) consisting of saponin QuilA adjuvant, phospholipids, and cholesterol	-	Enhances Th1 and Th2 responses. it is characterized by enhanced immune cell trafficking, induces central immune cell activation and maturation, and allows antigen dose reduction.	[135,147]
Matrix-M (Novavax)	Combined antigen delivery system, nanoparticles (40 nm) consisting of Matrix-A and –C at the ratio of 85:15. Matrix-A (Fraction-A, phospholipids, and cholesterol) and Matrix-C (Fraction-C, phospholipids, and cholesterol) Fraction-A and -C extracted from *Quillaja saponaria* bark.	Nuvaxovid/Novavax (anti-SARS-CoV-2, WHO, FDA and EMA approved for emergency uses)	Enhances Th1 and Th2 responses, and induces antibodies of multiple subclasses, enhances immune cell trafficking, induces central immune cell activation and maturation, and allows antigen dose reduction.	[148,149,150]

QS: Quillaja saponins; QS-21: *Quillaja saponaria* Molina, fraction 21 (Antigenics Inc., a wholly owned subsidiary of Agenus Inc., Lexington, MA, USA); Quil A: a fraction (mixture) of several different saponins from *Quillaja saponaria* Molina; MPL: 3-O-desacyl-4′-monophosphoryl lipid A, is the lipid A portion of LPS from *Salmonella minnesota* with agonist function on Toll-like receptor 4 (TLR4); CpG7909: an immunomodulating synthetic oligonucleotide designed to specifically agonise the Toll-like receptor 9 (TLR9).

Other saponin-based adjuvant systems have been proposed in order to improve efficacy and promote prolonged immunity with COVID-19 vaccines. Between these, adjuvant systems based on QS-21 have been widely suggested and studied as an alternative for boosting recombinant vaccines. These systems use the complete S protein as an immunogen [36,49,120], or its S1 domain [150] or only its receptor binding domain (RBD)/nucleocapsid (N) [122]. Currently, these adjuvant systems are being studied in clinical trials (i.e., still in progress), in which it is proposed to evaluate the safety, reactogenicity, and immune response against COVID-19 of a recombinant S protein-based vaccine with the QS-21 adjuvant (NCT04784767). In this context, the use of a varicella herpes zoster virus vaccine (Shingrix, adjuvanted with QS-21) has also been suggested for the purposes of the activation of the innate immune system against SARS-CoV-2 (NCT04523246).

## 4. Concluding Remarks

This review has critically examined the recent evidence on the emerging role of plant-derived saponins against the COVID-19 disease, highlighting the current understanding of the structure-activity relationship of these compounds. This is in addition to the molecular mechanisms of action against SARS-CoV-2 that emerge from in vitro, in vivo, and in silico models, as well as the clinical trials based on pulmonary models, which are proposed as antiviral, anti-inflammatory, antithrombotic, and immunostimulatory agents.

Among the saponins that have the anti-SARS-CoV-2 capacity, Glycyrrhizin, Escin, and Saikosaponins stood out. These molecules inhibited the cytopathic effects of the virus on cells through virus replication, but also virus adsorption and penetration into cells. Molecular docking analyses showed that their antiviral mechanisms of action were related to the inhibition of virus entries into the host cell (i.e., the binding and inhibition of human ACE2 or the viral S protein) or the inhibition of key processes in viral replication (inhibition of M^pro^ and Nsps).

In the same way, a wide variety of triterpene and steroidal saponins have been shown to exert anti-inflammatory effects on models of pulmonary inflammation, which has led to the proposal of these molecules as a complement to the therapy of severe patients with COVID-19. Specifically, with those who have developed symptoms of “Cytokine storm”, a hyperinflammatory condition that begins in the lung and then spreads, damaging different organs and causing death. Anti-inflammatory saponins include those from ginseng (including Ginsenoside Rg1, Rb1, Rg3, Rg5, Rh2, and Ro), Chai Hu (including Saikosaponin A and D), licorice (Glycyrrhizin), and horse chestnut (Dioscin), which were able to reduce histopathological changes associated with pulmonary inflammation. This is achieved by the downregulating of the expression and secretion of proinflammatory cytokines (TNF-α, IL-1β, IL-6 and IL-8), as well as the increasing of the expression of anti-inflammatory cytokines (TGF-β1 and IL-10) through different pathways (mainly by HMGB1, TLR4, MyD88, MAPK, and NF-κB-p65 signaling cascades). Additionally, different traditional medicines rich in ginseng saponins (with a high content of Notoginsenoside R1 and Ginsenoside Re, Rg1, Rb1, Rb2, Rb3, Rc, and Rd) have shown a great antiplatelet/antithrombotic capacity in models of pulmonary thromboembolism. Therefore, it has been proposed to use these compounds for the purposes of preventive treatment or complementary treatment to low molecular weight anticoagulants in different pulmonary thromboembolic diseases associated with severe COVID-19 patients (such as pulmonary thromboembolism, DIC, and PIC). The molecular mechanisms were associated with the inhibition of platelet activation and consequently the formation of thrombi, either by regulating the increase in cAMP and cGMP, mobilization, and reduction in the Ca^2+^ level. This is in addition to the inhibition of the secretion of granules with platelet agonists and inhibition of different coagulation factors.

On the other hand, the immunostimulant activity of several saponins has been highlighted, especially those from Quillaja saponaria (QS). These have the ability to stimulate both the Th1 and Th2 type immune responses and consequently cause an increase in specific antibodies (multiple subclasses) against antigens, the enhancement of immune cell trafficking, and the activation and maturation of central immune cells. QS have come to be used to enhance immunogenicity in different vaccines for intracellular pathogens. They can be used either in the form of a free adjuvant (e.g., QS-21) in formulations with other immunostimulants (e.g., AS01, AS02, and AS15), in complex immunostimulants (e.g., ISCOM), or in structures (e.g., Matrix-M1). Among the QS-based vaccines shown to be safe and effective—including the vaccine against the varicella herpes zoster virus (Shingrix^®^, approved by the FDA); the vaccine against the malaria parasite Plasmodium falciparum (Mosquirix^®^, approved by the EMA); and the one recently authorized for emergency use against COVID-19 (Nuvaxovid™, NVX-CoV2373)—a vaccine has been proposed as an alternative to better deal with the evolved variants of SARS-CoV-2 (which are characterized by having multiple mutations in the RBD region of the S protein). Due to this, the NVX-CoV2373 vaccine adjuvanted with Matrix-M was suggested. This is because it is composed of the complete S protein, which has different common epitopes with the different mutations present in the SARS-CoV-2 variants. It can also generate better titers of anti-S IgG antibodies and a better neutralization response against the different variants. However, more clinical studies are still needed in order to confirm and study the potential of saponins as adjuvants to, thus, improve the efficacy of anti-SARS-CoV-2 vaccines.

Finally, considering the limited therapeutic management of the COVID-19 disease, this review highlighted the promising use of saponins and their potential role in the new global scenario in the post-pandemic era. This is where there is scientific evidence that demonstrates their use as innovative natural molecules against SARS-CoV-2. This can be achieved either as antiviral agents exerting effects on different pharmacological targets of the virus, or as anti-inflammatory and antithrombotic agents relieving symptoms and clinical complications related to the disease, as well as immunostimulatory agents improving the efficacy and safety of vaccines in order to prolong immunogenicity against SARS-CoV-2 and its infectious variants.

## Figures and Tables

**Table 1:** Summary of the main studies related to the anti-SARS-CoV-2 activity of saponins and their effects on possible pharmacological targets.

Saponin [Chemical Structure]	Plant Source	Type of Study	Active Against	Main Findings	Doses	Mechanism of Action/Viral Target	Ref.
Saikosaponins A, B_2_, C and D [Triterpenoid saponins]	*Bupleurum falcatnum*	In vitro (Inhibition of cytopathic effects in MRC-5 cells by XTT cell-proliferative assay)	Human coronavirus HCoV-229E (ATCC)	All tested saikosaponins demonstrated antiviral activity. Saikosaponins A and B_2_ did not exhibit cytotoxic effects on MRC-5 cells at concentrations that achieved antiviral activity. The strongest activity was observed for Saikosaponin B_2_ (EC_50_: 1.7 μM), which significantly inhibited human coronavirus 229E infection by perturbing viral binding and penetration.	EC_50_ Saikosaponin A: 8.6 μM Saikosaponin B_2_: 1.7 μM Saikosaponin C: 19.9 μM Saikosaponin D: 13.2 μM	Disruption and Inhibition of virus-host cell binding by perturbation of viral glycoproteins.	[38]
Vernonioside (A_2_, A_4_ and D_2_) [Steroidal saponin]	*Vernonia amygdalina*	In silico (molecular docking: M^pro^-Vernonioside saponins interaction)	SARS-CoV-2	Vernonioside A_2_, Vernonioside A_4_ and Vernonioside D_2_ bound to catalytic dyad His41 and Cys145 residues located within the active sites and reported binding free energies of 8.6, −8.3, and −8.4 kcal/mol, respectively.	-	Binding to the active site of M^pro^ and inhibition of proteolysis of Nsps proteins necessary for viral replication.	[39]
XGG-pentahydroxycycloartane Ginsenoside Rg12 TPG1 [Triterpenoid saponins]	*Astragalus brachycalyx* *Panax ginseng* *Trevesia palmata*	In silico (molecular docking: M^pro^-triterpene saponin interaction)	SARS-CoV-2	XGG-pentahydroxycycloartane, Ginsenoside Rg12 and rhamnopyranosyl asiatic acid were the saponins with the highest potential for M^pro^ inhibition (−11.9, −10.9 y −10.9 kcal/mol). respectively), presentando mayor afinidad de unión que hydroxychloroquine (−4.6 kcal/mol), chloroquine (−5.6 kcal/mol), y nelfinavir (−7.6 kcal/mol).	-	Binding with M^pro^ and potential inhibition of proteolysis of Nsps proteins necessary for viral replication.	[40]
Arjunic acid Theasapogenil B Euscaphic acid [Triterpenoid saponins]	*Terminalia arjuna* *Camellia sasanqua* *Geum japonicum*	In silico (molecular docking: M^pro^-triterpene saponin interaction)	SARS-CoV-2	Arjunic acid, Theasapogenil B, and Euscaphic acid bind (hydrogen bonds, and alkyl and pi-alkyl interactions) to the Leu287 and Leu286 residues of the allosteric pocket of SARS-CoV-2 Mpro with binding free energies of −8.1, −8.1 and −8.0 kcal/mol, respectively. These saponins presented better pharmacokinetic and bioavailability properties than the N3 inhibitor of M^pro^, and also with Remdesivir and Dexamethasone.	Inhibition constant (K_i_), μM: Arjunic acid: 1.16 Theasapogenil B: 1.16 Euscaphic acid: 1.37 N3 inhibitor: 78.85 Remdesivir: 2.70 Dexamethasone: 2.28	Binding with the allosteric pocket of M_pro_ and inhibition of proteolysis of Nsps proteins necessary for viral replication.	[41]
Glycyrrhizin (GLR) [Triterpenoid saponin]	*Glycyrrhiza* spp.	In vitro (Inhibition of cytopathic effects in Vero cells by MTT cell-proliferative assay)	SARS-CoV (clinical isolates FFM-1 and FFM-2 from SARS patients)	GLR inhibited virus replication (EC_50_ of 300 µg/mL), but also virus adsorption and penetration into cells.	EC_50_ 300 µg/mL	Induction of nitric oxide synthase activity and increased nitric oxide exerting a toxic effect on the virus.	[56]
Glycyrrhizin [Triterpenoid saponin]	*Glycyrrhiza uralensis*	In vitro (plaque reduction assay on Vero cells)	SARS-CoV (clinical isolates from 10 SARS patients)	GLR inhibited virus replication (EC_50_ of 100 µg/mL), however, the rapid metabolization of the drug did not allow for achieving this effective concentration after intravenous administration of GLR 200 mg (80 µg/mL) to patients.	EC_50_ 100 µg/mL	Inhibition of virus entry into the host cell	[57]
Glycyrrhizin and amide derivatives or amino acid conjugates [Triterpenoid saponins]	*Glycyrrhiza glabra*	In vitro (Inhibition of cytopathic effects in Vero cells by MTT cell-proliferative assay and evaluation of viral antigens in membrane by immunocytochemistry)	SARS-CoV	The most active GLR derivatives were glycopeptide 2 containing S-benzyl-L-cysteine and glycopeptide 3 containing glycyl-L-leucine with antiviral activity 10 times greater than GLR. Those GLR amides and GLR conjugates with two amino acid residues, in addition to a free 30-COOH function, significantly increased activity against SARS-CoV, but also increased its cytotoxic effect.	EC_50_ Glycopeptide 2 (GLR conjugated to S-benzyl-L-cysteine): 35 µM Glycopeptide 3 (GLR conjugated to glycyl-L-leucine): 139 µM GLR: 365 µM	Binding of the N-acetylglycosamine residues to the carbohydrates of the SARS-CoV Spike glycoproteins, preventing binding to the ACE2 receptor and inhibiting the entry of the virus into the host cell.	[58]
Escin [Triterpenoid saponin]	*Aesculus hippocastanum*	In vitro (plaque reduction assay on Vero E6 cells)	SARS-CoV (H.K. strain)	Escin inhibited cytopathic effects and SARS-CoV replication reporting an EC_50_ of 6 μM	EC_50_ 6 µM	Inhibition of virus entry into the host cell.	[59]
Glycyrrhizin [Triterpenoid saponin]	*Glycyrrhiza* spp.	In silico (molecular docking: ACE2-glycyrrhizin interaction)	SARS-CoV-2	Glicirricina se une a ACE2 (−9.0 kcal/mol), interactuando con aminoácidos cercanos a la metalopeptidasa de zinc (Arg559, Gln388, Arg393 y Asp30) que podrían regular la actividad de ACE2.	-	Unión con la ACE2 humana e inhibición de la unión y entrada de SARS-CoV-2 en la célula huésped.	Chen y Du [60]
Glycyrrhizin Saikosaponin A [Triterpenoid saponins]	*Glycyrrhiza* spp. *Bupleurum falcatnum*	In silico (molecular docking: ACE2 and M^pro^-glycyrrhizin interaction)	SARS-CoV-2	GLR and Saikosaponin A showed higher binding affinity to the active sites of human ACE2 with a binding free energy of −9.9 and −11.0 kcal/mol, respectively, compared to M^pro^ from SARS-CoV-2 where they reported a free energy binding of −8.9 and −8.8 kcal/mol, respectively.	-	Binding to human ACE2 and inhibition of SARS-CoV-2 binding and entry into the host cell. Binding with M^pro^ and potential inhibition of proteolysis of Nsps proteins necessary for viral replication.	[61]
Saikosaponins (A, B_1_, B_2_, B_3_, B_4_, C, D, E, and I) [Triterpenoid saponins]	*Bupleurum falcatnum*	In silico (molecular docking: RBD site of glycoprotein Spike-Saikosaponins interaction)	SARS-CoV-2	Most Saikosaponins (except B_2_, C, and I) favorably bind to the RDB region of the Spike glycoprotein. Saikosaponin B_4_ was the best inhibitor with a binding free energy of −13.2 kcal/mol and interacting with the Asp428, Arg466, Glu465 and Phe464 residues of the RBD.	-	Binding to the RBD of the Spike viral protein and inhibition of binding of SARS-CoV-2 to host cell ACE2.	[62]
Saikosaponins (23 different molecules) [Triterpenoid saponins]	*Bupleurum scorzonerifolium*	In silico (molecular docking: glycoprotein Spike and Nsp15-Saikosaponins interaction)	SARS-CoV-2	Saikosaponins U and V showed better binding with the pharmacological targets under study. Saikosaponin U had a better affinity for binding (H-bond) with the RBD of the S1 subunit of the Spike glycoprotein, positioning itself in the N-terminal domain (residues 319–519) with a free binding energy of −8.4 kcal/mol. Saikosaponin V had a better affinity for binding (H-bond) to the Lys290, Thr341, Tyr343 and Ser294 residues of the Nsp15 active site, with a binding free energy of −8.4 kcal/mol.	-	Binding to the RBD of the Spike viral protein and inhibition of binding of SARS-CoV-2 to ACE2 of host cells. Binding to Nsp15 and inhibition of its activity in the RTC complex for viral RNA synthesis and replication.	[63]

## Data Availability

Not applicable.

## Supplementary Materials

The following supporting information can be downloaded at: https://www.mdpi.com/article/10.3390/pharmaceutics15020348/s1, Table S1: Summary of the main articles related to the anti-inflammatory effects of saponins studied in in vivo models of pulmonary inflammation; Table S2: Summary of the main studies related to the antiplatelet-antithrombotic effects of saponins in clinical trials and in vivo models of pulmonary coagulopathies.
