# Supplementary material for: Saponins: Research Progress and Their Potential Role in the Post-COVID-19 Pandemic Era
Source: Pharmaceutics. 2023 Jan 20;15(2):348. doi: 10.3390/pharmaceutics15020348 (PMC9964560; doi:10.3390/pharmaceutics15020348)
Supplement: Supplementary file 1 [file pharmaceutics-15-00348-s001.zip › pharmaceutics-2082979-supplementary.pdf]

pharmaceutics

MDPI

Review

Saponins: Research progress and their potential role in the post-COVID-19 pandemic era

Daniel Mieres-Castro [1] and Freddy Mora-Poblete [1,*]

[1] Institute of Biological Sciences, University of Talca, 1 Poniente 1141, Talca 3465548, Chile
* Correspondence: morapoblete@gmail.com; Tel.: +56 71 2418878

Supplementary Materials:

Table S1. Summary of the main articles related to the anti-inflammatory effects of saponins studied in in vivo models of pulmonary inflammation.

Saponin [chemical structure]	Plant source	Type of Study	Activity	Main Findings	Doses	Mechanism of Action	Ref.
Saikosaponin A [Triterpenoid saponin]	*Radix Bupleuri* (*Bupleurum chinense* and *B. scorzonerifolium*)	In vivo (ALI induced by infection with influenza A in mice)	Anti-inflammatory	Significant inhibition of viral replication, abnormal production of proinflammatory cytokines (IFN-γ, TNF-α, IL-1β, and IL-6), and pulmonary histopathology. Significant inhibition of signaling of the NF-κB pathway. Selective attenuation of the recruitment of pulmonary neutrophils and monocytes.	25 mg/kg	Inhibition of the NF-κB signaling pathway and selective attenuation of the recruitment of pulmonary neutrophils and monocytes	[53]
PNS (Yunnan Phytopharmaceutical Co. Ltd., Kunming, China)	*Panax notoginseng*	In vivo (acute lung injury (ALI) induced by oleic acid and lipopolysaccharide in rats)	Anti-inflammatory	Significant reduction in lung parenchyma damage and extravascular lung water content. Significant decrease in total leukocyte and neutrophil counts, proinflammatory cytokines (TNF-α and IL-6), and the αENaC channel (protein and mRNA).	100 mg/kg	Restoration of protein and mRNA expression of αENaC and through anti-inflammatory effects	[73]

PNS<br>(Kunming Pharmaceutical Group<br>Co., Ltd., Yunnan, China)	*Panax notoginseng*	In vivo (pulmonary fibrosis in rabbits)	Anti-inflammatory	Significant decrease in lung injury and expression levels of AST, LDH, CK, IL-6 and IL-8.<br>Significantly decreased activation of NF-κB (decreased expression of NF-κB-p65).	50 mg/kg daily, for 28 days	Inhibition of the NF-κB signaling pathway	[74]
Ginsenoside Rg3<br>[Triterpenoid saponin]	*Panax notoginseng*	In vivo (ALI induced by omethoate in rats)	Anti-inflammatory	Significant increase in glutathione (GSH) content in the lung.<br>Significant decrease in the content of malondialdehyde (MDA), TNF-α.<br>Significant decrease in the activity of the enzymes superoxide dismutase (SOD), catalase (CAT) and myeloperoxidase (MPO).	5, 10, or 20 mg/kg	Decreased oxidative stress by increasing reduced glutathione content and decreasing the activity of SOD, CAT and myeloperoxydase enzymes	[75]
Ginsenoside Rg1<br>[Triterpenoid saponin]	*Panax notoginseng*	In vivo (ALI induced by sepsis in mice)	Anti-inflammatory	Significant decrease in lung damage, and survival rate.<br>Significant suppression of the secretion of inflammatory cytokines (TNF-α and IL-6).<br>Significant increase in the expression of sirtuin 1 (SIRT1)	10 or 20 mg/kg	Decreased oxidative stress by increasing the expression of sirtuin 1 (SIRT1) in the endoplasmic reticulum	[76]

Ginsenoside Rb1 [Triterpenoid saponin]	*Panax notoginseng*	In vivo (ALI induced by Staphylococcus aureus in mice)	Anti-inflammatory	Significant attenuation of lung injury. Significant inhibition of the production of IL-1β, IL-6 and TNF-α. Significant inhibition of TLR2 receptor activation and NF-kB and MAPK signaling (decreased phosphorylation of NF-κB-p65, ERK and JNK).	10 or 20 mg/kg	TLR2-mediated inhibition of the proinflammatory NF-κB and MAPK signaling pathways	[77]
Ginsenoside Rg1 [Triterpenoid saponin]	*Panax notoginseng*	In vivo (ALI induced by ischemia reperfusion in rats)	Anti-inflammatory	Significant attenuation of histological abnormalities in the lung. Significant decrease in the 6-keto-PGF1α/TXB2 ratio, and MPO activity. Significant inhibition of NF-κB activation and decreased COX-2 expression in lung tissue	40 mg/kg	Inhibition of the NF-κB/COX-2 signaling pathway	[78]
Ginsenoside Rg3 [Triterpenoid saponin]	*Panax notoginseng*	In vivo (ALI induced by lipopolysaccharide (LPS) in mice)	Anti-inflammatory	Significant reduction in pathological damage. Significant decrease in MPO activity, proinflammatory cytokines (TNF-α), IL-1β and IL-6) Significant increase in anti-inflammatory mediators (IL-10	10, 20, or 30 mg/kg	Inhibition of the PI3K/Akt/mTOR pathway dependent on MerTK activation	[79]

				and TGF-β), M2 macrophage polarization, and phosphorylated MerTK expression levels<br>Significantly decreased PI3K, Akt, and mTOR phosphorylation			
Ginsenoside Rg5 [Triterpenoid saponin]	*Panax notoginseng*	In vivo (ALI induced by LPS in mice)	Anti-inflammatory	Significant inhibition of the expression of proinflammatory cytokines (IL-1β and TNF-α), inflammatory enzymes (COX-2 and iNOS) and activation of the NF-κB pathway in bronchoalveolar lavage fluid (BALF) and in alveolar macrophages.<br>Significant inhibition of the interaction between LPS and TLR4 in alveolar macrophages	2.5, 5 or 10 mg/kg	Inhibition of the TLR4 signaling pathway in macrophages and lung tissue	[80]
Ginsenoside Rh2 [Triterpenoid saponin]	*Panax notoginseng*	In vivo (ALI induced by LPS in mice)	Anti-inflammatory	Significant decrease in histological changes in lung tissues and BALF protein content.<br>Significant increase in the expression of HO-1, Nrf-2, CAT, SOD and GPx in lung tissue.<br>Significantly decreased production of NO, TNF-α, IL-1β, IL-4, IL-6 in lung tissues.	5, 10, and 20 mg/kg	Regulation of TLR4/PI3K/Akt/mTOR, Raf-1/MEK/ERK and Keap1/Nrf2/HO-1 signaling pathways	[81]

				Significant inhibition of the expression of iNOS, COX-2, and the phosphorylation of IκBα, ERK, JNK, p38, Raf-1 and MEK.			
Ginsenoside Ro<br>[Triterpenoid saponin]	*Panax notoginseng*	In vivo (ALI induced by LPS in mice)	Anti-inflammatory	Significant reduction in lung injury.<br>Significant inhibition of the expression and secretion levels of proinflammatory cytokines (TNF-α, IL-1β and IL-6).<br>Significant inhibition of activation of the NF-κB and MAPK pathways<br>Ginsenoside Ro coupled to the LPS binding site of the TLR4/MD2 complex	20, 40, and 80 mg/kg	Inhibition of the TLR4 signaling pathway in macrophages and lung tissue	[82]
Saikosaponin D<br>[Triterpenoid saponin]	*Radix Bupleuri* (*Bupleurum chinense and B. scorzonerifolium*)	In vivo (ventilator-induced lung injury in rats)	Anti-inflammatory	Significantly decreased expression of proinflammatory cytokines (MIP-2, IL-6 and TNF-α) and oxidative enzymes (MPO)<br>Significant increase in the expression of anti-inflammatory mediators (TGF-β1 and IL-10).<br>Significant decrease in apoptosis (Caspase-3 and Bax) and	80 mg/kg	Inhibition of oxidative stress and apoptosis in lung tissue	[83]

				increase in anti-apoptotic proteins (bcl-2)			
Saikosaponin A<br>[Triterpenoid saponin]	*Radix Bupleuri* (*Bupleurum chinense and B. scorzonerifolium*)	In vivo (ALI induced by LPS in mice)	Anti-inflammatory	Significant reduction in lung injury.<br>Significant inhibition of MPO activity and inflammatory cytokines (TNF-α and IL-1β) in BALF<br>Significant inhibition of NF-κB pathway activation and NLRP3 inflammasome expression.	5, 10, or 20 mg/kg	Inhibition of the NF-κB and NLRP3 signaling pathway	[84]
Glycyrrhizin<br>[Triterpenoid saponin]	*Glycyrrhiza* spp.	In vivo (ALI induced by LPS in mice)	Anti-inflammatory	Significant decrease in the expression of TLR2, inflammatory cells, collagen, MIP-2, KC, IL-4, IL-6, GM-CSF and IFN-γ.<br>Significant inhibition of the TLR signaling pathway and the NF-κB signaling pathway.	0.5mg/mL<br>(In respiratory tract)	Inhibition of the TLR2 signaling pathway	[85]
Glycyrrhizin<br>[Triterpenoid saponin]	*Glycyrrhiza* spp.	In vivo (ALI induced by LPS in mice)	Anti-inflammatory	Significant decrease in histopathological changes in the lung.<br>Significant decrease in TLR4 and CXCR4 in lung tissue<br>Significant decrease in<br>inflammatory cell count, pro-inflammatory cytokines	50 mg/kg, twice daily	Inhibition of the TLR4/NF-κB signal pathway	[86]

				(TNF-α, IL-1β, and IL-6), MPO activity, and expressions of COX-2, iNOS, and NF-Kb in BALF.			
Glycyrrhizin [Triterpenoid saponin]	*Glycyrrhiza* spp.	In vivo (ALI induced by radiation in mice)	Anti-inflammatory	Significant decrease in plasma concentrations of HMGB1, sRAGE, and expression levels of proinflammatory cytokines (TNF-α, IL-1β and IL-6) in BALF. Significant inhibition of downstream transcription factors related to the HMGB/TLR4 pathway (NF-κB, JNK and ERK1/2) in lung tissue.	10 mg/kg three times per week until week 12	Inhibition of the HMGB1/TLR4 signal pathway	[87]
Glycyrrhizin [Triterpenoid saponin]	*Glycyrrhiza* spp.	In vivo (ALI induced by LPS in mice)	Anti-inflammatory	Significant decrease in lung injury Significantly decreased production of inflammatory factors TNF-α, IL-1β and HMGB1. Significant increase in autophagy (increased number of autophagosomes) through upregulation of LC3-II/I and Beclin-1 protein levels and downregulation of SQSTM1/P62.	200 mg/kg	Regulation of autophagy related to the negative regulation of the PI3K/Akt/mTOR pathway	[88]

				Glycyrrhizin-induced autophagy was associated with negative regulation (decreased phosphorylation levels) of the PI3K/Akt/mTOR pathway.			
Esculentoside A [Triterpenoid saponin]	*Phytolacca esculenta*	In vivo (ALI induced by LPS in mice)	Anti-inflammatory	Significant decrease in inflammatory infiltration, alveolar wall thickening, and pulmonary congestion<br>Significant decrease in proinflammatory cytokines (TNF-α and IL-6) in BALF.<br>Significantly decreased phosphorylation of IκBα, p38 and ERK.	15, 30, and 60 mg/kg	Inhibition of NF-κB and MAPK signaling pathways	[89]
Anemoside B4 [Triterpenoid saponin]	*Pulsatilla chinensis*	In vivo (ALI induced by LPS in mice)	Anti-inflammatory	Significant decrease in lung damage.<br>Significant decrease in the CD4+/CD8+ T lymphocyte ratio<br>Significantly decreased phosphorylation of NF-κB-p65 and IκBα	12.5, 25, 50 mg/kg	Inhibition of NF-κB signaling pathways	[90]
Astragaloside IV [Triterpenoid saponin]	*Astragalus membranaceus*	In vivo (ALI induced by paraquat in mice)	Anti-inflammatory	Significant decrease in histopathological changes in the lung.<br>Significant decrease in the levels of MDA, MPO and	50 mg/kg or 100 mg/kg daily for 5 days	Trx/Txnip regulation and inhibition of Rho/ROCK/	[91]

				proinflammatory cytokines (TNF-α, IL-1β and IL-6).<br>Significant increase in SOD, CAT and GSH-Px levels.<br>Significantly decreased expression of Txnip/Trx and the Rho/ROCK/NF-κB signaling pathway.		NF-κB signals	
Asiaticoside<br>[Triterpenoid saponin]	*Centella asiatica*	In vivo (septic lung injury<br>in mice)	Anti-inflammatory	Significant decrease in histopathological changes in the lung.<br>Significant inhibition of COX-2 and iNOS expression, and increased expression of PPAR-γ in lung tissues.<br>Significant decrease in TNF-α and IL-6 levels.<br>Significant inhibition of ERK1/2, JNK, P38 and IκBα phosphorylation.	45 mg/kg	Inhibition of the NF-κB and MAPK signaling pathways mediated by PPAR-γ	[92]
Asiaticoside<br>[Triterpenoid saponin]	*Centella asiatica*	In vivo (ALI induced by LPS in mice)	Anti-inflammatory	Significant decrease in inflammatory infiltration and histopathological changes in the lung.<br>Significantly decreased MPO activity and TNF-α and IL-6 levels in BALF.	15, 30 or 45mg/kg	Inhibition of NF-κB signaling pathways	[93]

				Significant increase in IκBα expression and decrease in NF-κB-p65 phosphorylation.			
Tenuigenin<br><br>[Triterpenoid saponin]	*Polygala tenuifolia*	In vivo (ALI induced by LPS in mice)	Anti-inflammatory	Significant decrease in histopathological changes in the lung.<br><br>Significant inhibition of<br><br>Significant decrease in MPO activity in lung tissue.<br><br>Significantly decreased COX-2 expression and TNF-α, IL-1β and IL-6 levels in BALF.<br><br>Significantly decreased phosphorylation of IκBα, p38 and ERK.	2, 4 or 8 mg/kg	Inhibition of NF-κB and MAPK signaling pathways	[94]
Pseudoginsenoside-F11<br><br>[Triterpenoid saponin]	*Panax pseudoginseng* subsp. *Himalaicus*	In vivo (ALI induced by LPS in mice)	Anti-inflammatory	Significant decrease in histopathological changes in the lung.<br><br>Significantly decreased expression of<br><br>TNF-α, IL-1β and IL-6 in lung tissue and BALF.<br><br>Significantly decreased expression of<br><br>MIP-2 and ICAM-1 and neutrophil infiltration	3, 10,or 30 mg/kg	Suppression of neutrophil infiltration and acceleration of neutrophil clearance	[95]

				Significant increase in neutrophil clearance in BALF.			
Platycodin D<br>[Triterpenoid saponin]	*Platycodon grandiflorum*	In vivo (ALI induced by LPS in mice)	Anti-inflammatory	Significantly decreased pathological changes and the wet to dry weight (W/D) ratio of the lungs.<br>Significant decrease in total leukocyte number and percentage of neutrophils in BALF and MPO activity in lung tissue.<br>Significantly decreased TNF-α and IL-6 levels and increased SOD activity in BALF.<br>Significantly decreased expression of NF-κB-p65, Caspase-3 and Bax, and increased expression of Bcl-2 in lung tissue.	50 mg/kg or 100 mg/kg	Suppression of apoptosis by downregulation of Caspase-3 and Bax	[96]
Platycodin D<br>[Triterpenoid saponin]	*Platycodon grandiflorum*	In vivo (ALI induced by LPS in mice)	Anti-inflammatory	Significant decrease in histopathological changes and MPO activity in the lungs.<br>Significantly decreased macrophage and neutrophil, and TNF-α, IL-1β, and IL-6 levels in BALF.	20, 40, or 80 mg/kg	Positive regulation of the LXRα-ABCA1 signaling pathway	[97]

				Significantly decreased phosphorylation of NF-κB-p65 and IRF3. Significant decrease in lipid raft formation (GM-1 expression) and TLR4 recruitment. Significant decrease in cholesterol levels in membrane lipid rafts Significant increase in ABCA1 transcriptional activity and LXR*α* expression.			
Escin [Triterpenoid saponin]	*Aesculus hippocastanum*	In vivo (ALI induced by endotoxin in mice)	Anti-inflammatory	Significant decrease in histopathological changes in the lung. Significant decrease in MPO activity and serum MDA, NO, TNF-*α*, IL-1β and IL-6 levels. Significant increase in SOD and GPx activity in serum. Significant increase in protein expression of glucocorticoid receptors (GR)	0.9, 1.8 or 3.6 mg/kg	Increased expression of GR and endogenous antioxidant capacity	[98]
Escin [Triterpenoid saponin]	*Aesculus hippocastanum*	In vivo (ALI induced by LPS in rats)	Anti-inflammatory	Significant decrease in histopathological changes in the lung. Significantly decreased levels of proinflammatory cytokines	Monotherapy: Escin 5 mg/kg	Negative regulation of the signaling cascades HMGB1,	[99]

				(TNF-α, IL-1β, and IL-6) in serum, BALF, and lung tissue.<br>Significant decrease in inflammasomes (NLRP3 expression) and pyroptosis.<br>Significantly decreased expression of HMGB1, TLR4, MyD88, MAPK, and NF-κB-p65.<br>Combined treatment (Escin + Q10) showed better results in all trials (synergistic effect).	Combined treatment: Escin<br>(5 mg/kg) + coenzyme Q10 (CoQ10) (100 mg/kg)	TLR4, MyD88, MAPK and NF-κB-p65.	
Dioscin<br>[Steroidal saponin]	*Dioscorea* spp.	In vivo (pulmonary fibrosis induced by crystalline silica in mice)	Anti-inflammatory	Significant decrease in histopathological changes and the infiltration of macrophages, B lymphocytes, T lymphocytes and fibrocytes in the lung.<br>Significantly decreased levels of TNF-α, TGF-β, IFN-γ, IL-1β, IL-2, IL-4, IL-6, IL-13, IL-17A in BALF and lung tissue.<br>Significantly decreased ASK-1, p38 and JNK phosphorylation.	20, 40 or 80 mg/kg	Inhibition of the ASK-1-p38/JNK signaling pathway	[100]
Dioscin<br>[Steroidal saponin]	*Dioscorea* spp.	In vivo (ALI induced by bleomycin in mice)	Anti-inflammatory	Significant decrease in histopathological changes, MPO activity and MDA contents in lung tissue.	80 mg/kg	Inhibition of the NF-κB signaling pathway	[101]

				Significant decrease in the total number of cells, macrophages, neutrophils, and total protein in BALF.<br>Significantly decreased expression (mRNA and protein) of TNF-α, IL-1β and IL-6 in lung tissue.<br>Significantly decreased protein expression of HGMB1, COX-2, and NF-κB-p65 in lung tissue.			
Dioscin<br>[Steroidal saponin]	*Dioscorea* spp.	In vivo (ALI induced by LPS in mice and rats)	Anti-inflammatory	Significant decrease in histopathological changes in the lung.<br>Significant inhibition of inflammatory cell infiltration and MDA, SOD, NO and iNOS levels.<br>Significantly decreased levels of TNF-α, IL-1β, and IL-6 mRNA.<br>Significant decrease in protein levels of TLR4, MyD88, TRAF6, TKB1, TRAF3, and phosphorylation levels of PI3K, Akt, IκBα, NF-κB-p65.	20, 40, and 80 mg/kg for mice<br>15, 30, and 60 mg/kg for rats	Inhibition of the TLR4/MyD88 signaling pathway	[102]

Dioscin [Steroidal saponin]	*Dioscorea* spp.	In vivo (ALI induced by LPS in mice)	Anti-inflammatory	Significant decrease in lesion and water content in the lung. Significantly decreased total number of alveolar macrophages and total protein in BALF. Significantly decreased levels of TNF-α, IL-1β, IL-6, MPO, IFN-γ, ICAM-1, and NF-κB-p65. Significant decrease in protein expression of TLR4, MyD88, NF-κB-p65, COX-2 and HSP70.	20, 40, and 60 mg/kg	Inhibition of the TLR4/MyD88 signaling pathway	[103]
Dioscin [Steroidal saponin]	*Dioscorea* spp.	In vivo (ALI induced by LPS in mice)	Anti-inflammatory	Significant decrease in histopathological changes, permeability, edema and MPO activity in the lungs. Significantly decreased total cell and neutrophil numbers, total protein, TNF-α, IL-6, and keratinocyte chemoattractant (KC) in BALF. Significantly decreased protein expression of COX-2, TLR4, and phosphorylated NF-κB-p65.	20, 40, or 80 mg/kg	Inhibition of the TLR4/MyD88 signaling pathway	[104]

Dioscin [Steroidal saponin]	*Dioscorea* spp.	In vivo (Asthma induced by ovalbumin in mice)	Anti-inflammatory	Significant decrease in the infiltration of inflammatory cells in lung tissue. Significantly decreased levels of TNF-α, IL-1β and IL-6 in BALF. Significant increase in the expression (mRNA and protein) of α-glucocorticoid receptors (SLPI, GILZ and MKP-1) and inhibition of HSP70.	100 mg/kg	Increased expression of α-glucocorticoid receptors and inhibition of HSP70	[105]
Dioscin [Steroidal saponin]	*Dioscorea* spp.	In vivo (pulmonary fibrosis induced by crystalline silica in mice)	Anti-inflammatory	Significant decrease in inflammatory cell infiltration and fibrosis in lung tissue. Significant decrease in apoptotic cells (annexin A5 expression) and increase in autophagosomes by cells and lung tissue. Significant increase in LC3-II/I expression. Significantly decreased expression of p62 and phosphorylation of Akt and mTOR	20, 40 or 80 mg/kg	Increased autophagy of alveolar macrophages associated with the negative regulation of the Akt/mTOR pathway	[106]
Trillin [Steroidal saponin]	*Dioscorea nipponica*	In vivo (ALI induced by LPS in mice)	Anti-inflammatory	Significant decrease in pathological changes, MPO activity and W/D ratio in lung tissue.	50 or 100 mg/kg	Activation of the Nrf2/HO-1 signaling pathway and inhibition of	[107]

				Significant decrease in the levels of IL-1β and IL-6 and increase in the levels of SOD, MDA, CAT, GSH, GSH-px in BALF.<br><br>Significant increase in protein expression of Nrf-2 and HO-1 and decreased phosphorylation of NF-κB-p65 and IκBα		the NF-κB pathway	
Timosaponin A-III<br><br>[Steroidal saponin]	*Anemarrhena asphodeloides*	In vivo (ALI induced by LPS in mice)	Anti-inflammatory	Significant decrease in histopathological changes and levels of IL-1β and IL-6 in lung tissue.<br><br>Significant decrease in the total number of cells, macrophages and neutrophils in BALF.<br><br>Significantly decreased NF-κB-p65, p38, JNK, ERK, and STAT3 phosphorylation in lung tissue.	25 or 50 mg/kg	Inhibition of NF-κB and MAPK signaling pathways	[108]

Table S2. Summary of the main studies related to the antiplatelet-antithrombotic effects of saponins in clinical trials and in vivo models of pulmonary coagulopathies.

Saponin [chemical structure]	Plant source	Type of Study	Activity	Main Findings	Doses	Mechanism of Action	Ref.
extracts of total steroidal saponins (TSS)<br><br>protodeltonin, deltonin, parvifloside, y zingiberensis saponin<br><br>[Steroidal saponins]	*Dioscorea zingiberensis*	Ex vivo (platelet aggregation in rat model)<br><br>In vivo (bleeding time, coagulation factors, and protection rate in inferior vena cava ligation thrombosis rat model and pulmonary thrombosis mice model)	Anti-thrombotic	TSS-DZW significantly inhibited (up to 25% inhibition) ADP-induced platelet aggregation and reduced thrombus size induced by inferior vena cava ligation in rats by up to 84%.<br><br>TSS-DZW prolonged the time of coagulation parameters (APTT, TT, and PT) and bleeding time in a dose-dependent manner in mice.<br><br>TSS-DZW provided significant (45.4%) protection against death from pulmonary thrombosis in mice.	Oral administration for 2 weeks of TSS:<br>Rats: 32.3, 64.7, and 129.4 mg/kg<br>Mice: 45.3, 90.6, and 181.1 mg/kg	Inhibition of platelet aggregation	**[109]**
Naodesheng (NDS) formula<br><br>Notoginsenoside R1 y Ginsenoside (Re, Rg1, Rb1, Rb2, Rb3, Rc, y Rd)	*Rhizoma Chuanxiong, Lobed Kudzuvine, Carthamus tinctorius, Radix Notoginseng* and *Crataegus pinnatifida*	Ex vivo (platelet aggregation rat model)<br><br>In vivo (Blood clotting time and protection rate in	Anti-thrombotic	The NDS bioactive fraction CD significantly inhibited (22.48% inhibition) ADP-induced platelet aggregation in rats.<br><br>CD-NDS prolonged clotting time to and provided significant (60%) protection against	Daily oral administration of 2.14 g/kg for 5 days (rats and mice)	Inhibition of platelet aggregation	**[110]**

[Triterpenoid saponins]		pulmonary thrombosis mice model)		death from pulmonary thrombosis in mice.			
Diosgenyl β-D-galactopyranosyl-(1→4)-β-D-glucopyranoside (C3)<br>(diosgenin derivative)<br>[Steroidal saponin]	*Dioscorea zingiberensis*	Ex vivo (platelet aggregation in rat model)<br>In vivo (bleeding time, coagulation factors, and protection rate in pulmonary thrombosis mice model)	Anti-thrombotic	Compound C3 at a dose of 100 mg/kg significantly inhibited (15% inhibition) ADP- and thrombin-induced platelet aggregation in rats.<br>C3 increased APTT (20.95 sec), inhibited factor VIII activities (35% inhibition), and provided significant protection (45.45%) against death from pulmonary thrombosis in mice.	Oral administration of 25-100 μM, twice a day for five days (rats and mice)<br>In protection rate in pulmonary thrombosis mice model: oral administration of 25-100 mg/kg	Inhibition of platelet aggregation and factor VIII activities	**[111]**
Panax notoginseng saponins (PNS)<br>ginsenoside Rg1, ginsenoside Rb1 [Triterpenoid saponins]<br>notoginsenoside R1 [Steroidal saponin]	*Panax notoginseng*	In vitro (platelet activation and aggregation assays)<br>In vivo (improvement of the hypercoagulable state in rat hypercoagulable model)	Anti-platelet/ Anti-thrombotic	In in vitro assays, treatment with PNS (100 μg/mL) significantly inhibited thrombin-induced platelet aggregation (up to 20%) and was associated with PPAR-γ overexpression, its positive regulation of the PI3K)/Akt/ eNOS.<br>In in vivo trials, the doses of PNS (100 and 200 mg/kg) significantly reversed the hypercoagulability state induced in	In vitro assays: 1, 10, 100 μg/mL<br>In vivo assay: 10, 100 and 200 mg/kg	Inhibition of platelet activation and aggregation by upregulating the PI3K/Akt/eNOS pathway in a PPAR-γ-dependent manner	**[112]**

				rats, by prolonging the APTT and PT parameters (values similar to normal control) and decreasing the expression of fibrinogen.			
PNS from Xuesaitong Oral Tablets (Chinese Patent Medicine)<br><br>notoginsenoside R1 [Steroidal saponin]<br><br>ginsenoside Rg1, ginsenoside Rb1 [Triterpenoid saponins]	*Panax notoginseng*	Clinical trial (prospective cohort study, 281 surgical inpatients at moderate to high risk of DVT)	Anti-thrombotic	Significant decrease in the incidence of DVT in the group of exposure patients (PNS+LMWH) with 21 (15.7%) incidents of DVT, compared to the group of control patients (LMWH) with 41 (27.9%) incidents of DVT.	Control group: hypodermic injection of LMWH (4000–8000 AxaIU, once daily)<br><br>Exposure group: Xuesaitong oral tablets (100 mg), 3 times daily, combined with LMWH	Inhibition of platelet aggregation	**[113]**
